# No bidirectional relationship between sleep phenotypes and risk of proliferative diabetic retinopathy: a two-sample Mendelian randomization study

**DOI:** 10.1038/s41598-024-60446-3

**Published:** 2024-04-26

**Authors:** Huan Liu, Lin Li, Xiaoning Zan, Jing Wei

**Affiliations:** Department of Ophthalmology, The First Affiliated Hospital of Henan University of Science and Technology, No. 24 Jinghua Road, Luoyang, 471003 Henan People’s Republic of China

**Keywords:** Diabetic retinopathy, Sleep phenotypes, Mendelian randomization study, Single nucleotide polymorphisms, Causal relationship, Epidemiology, Genetics research, Endocrine system and metabolic diseases, Eye diseases, Genetic association study

## Abstract

This study aimed to investigate the probable existence of a causal relationship between sleep phenotypes and proliferative diabetic retinopathy (PDR). Single nucleotide polymorphisms associated with sleep phenotypes were selected as instrumental variables at the genome-wide significance threshold (*P* < 5 × 10^−8^). Inverse‐variance weighted was applied as the primary Mendelian randomization (MR) analysis method, and MR Egger regression, weighted median, simple mode, and weighted mode methods were used as complementary analysis methods to estimate the causal association between sleep phenotypes and PDR. Results indicated that genetically predicted sleep phenotypes had no causal effects on PDR risk after Bonferroni correction (*P* = 0.05/10) [Chronotype: *P* = 0.143; Daytime napping: *P* = 0.691; Daytime sleepiness: *P* = 0.473; Insomnia: *P* = 0.181; Long sleep duration: *P* = 0.671; Morning person:*P* = 0.113; Short sleep duration: *P* = 0.517; Obstructive sleep apnea: *P* = 0.091; Sleep duration: *P* = 0.216; and snoring: *P* = 0.014]. Meanwhile, there are no reverse causality for genetically predicted PDR on sleep phenotypes [Chronotype: *P* = 0.100; Daytime napping: *P* = 0.146; Daytime sleepiness: *P* = 0.469; Insomnia: *P* = 0.571; Long sleep duration: *P* = 0.779; Morning person: *P* = 0.040; Short sleep duration: *P* = 0.875; Obstructive sleep apnea: *P* = 0.628; Sleep duration: *P* = 0.896; and snoring: *P* = 0.047]. This study’s findings did not support the causal effect of between sleep phenotypes and PDR. Whereas, longitudinal studies can further verify results validation.

## Introduction

Diabetic retinopathy (DR) is the most common microvascular complication of diabetes and the leading cause of vision loss and blindness globally, with an overwhelming 103.12 million people with DR in 2020, which is envisaged to rise to 160.50 million by 2045^1,2^. DR is classified into two main clinical stages: non-proliferative diabetic retinopathy (NPDR) and proliferative diabetic retinopathy (PDR)^3^.

The pathogenesis of DR still needs to be better understood. Previous studies showed that retinal microvascular damage leading to vascular leakage and ischemia-induced retinal neovascularization plays an essential role in DR pathogenesis^4,5^. Hypertension, obesity, blood glucose level, glycated hemoglobin (Hb)A1c, hyperlipidemia, dietary style, exercise, and smoking are also involved in its development^6,7^. In addition, growing epidemiological evidence suggests that sleep disorders are becoming emerging risk factors DR^8^.

For example, obstructive sleep apnea (OSA), the most common type of sleep disorder, is characterized by repeated apneas and hypopneas during sleep, leading to hypoxia and hypercapnia, where, OSA was reported to be associated with an increased DR risk^9–11^. Similarly, both short (≤ 5 h/day) and long (≥ 9 h/day) sleep durations were also associated with an increased risk of DR in men^12^. However, studies investigating the role of OSA in DR have yielded conflicting results, where some studies studies even showed no association between OSA and DR^13,14^. Besides, considering the influence of selection biases, residual confounding, and reverse causality in observational studies, the causal effect between sleep disorders and the risk of DR is unclear.

Furthermore, the role of other sleep phenotypes such as daytime napping, sleepiness, chronotype, morning person, snoring, insomnia, sleep duration, short sleep duration, and long sleep duration in DR has yet to be extensively investigated. Revealing the causality of differential sleep phenotypes on DR might provide novel insights into the pathogenesis of diseases and devise future intervention studies.

Mendelian randomization (MR) is an emerging epidemiological method that uses genetic variants as instrumental variables to evaluate the causal relationship between exposures and outcomes and could reduce biases, residual confounding, and reverse causality^15^.

In the present study, to investigate the probable existence of a causal association between sleep phenotypes and PDR, a two-sample MR was performed to evaluate the associations between genetically predicted sleep phenotypes and PDR risk.

## Methods

### MR study design

**Figure 1:**
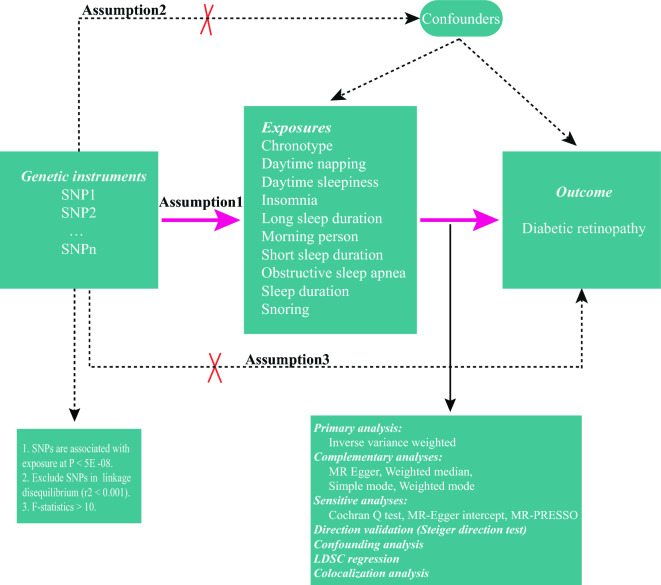
The overview of the MR study design. MR study is based on three main assumptions. Assumption 1: The genetic variants selected as instrumental variables should be associated with the risk factor. Assumption 2: Genetic variants used as instrumental variables should not be associated with known confounding factors. Assumption 3: The used genetic variants as instrumental variables should influence the risk of the outcome only via exposure.

A two-sample MR method was designed to estimate the magnitude of a causal effect of sleep phenotypes on PDR by using genetic variants as instrumental variables^16^. The overview of the study design is displayed in Fig. 1.

### Data for exposure

The summary-level data used in the study were obtained from publicly available GWASs of European ancestry (Table 1). The GWAS data on self-reported daytime napping, and sleepiness, chronotype, morning person, insomnia, sleep duration (7–9 h/day), short sleep duration (< 7 h/day), and long sleep duration (> 9 h/day) were download from the Sleep Disorder Knowledge Portal website (https://sleep.hugeamp.org/datasets.html)^17–21^. The GWAS data on obstructive sleep apnea and snoring were derived from FinnGen (Risteys R9)^22^, and MRC Integrative Epidemiology Unit (IEU) (GWAS ID : ebi-a-GCST009761), respectively^23,24^.

**Table 1:** The summary-level data used in the study.

Exposure and outcome	Consortium	Category	Cases	Controls	Total	Used SNPs	VEP	F statistic	PMID	DOI/URL
Chronotype	UK Biobank	CV	449,734	–	449,734	122	1.264	46.591 (28.834–209.387)	3069 6823	https://personal.broadinstitute.org/ryank/sleepdurationsumstats.txt.zip
Daytime napping	UK Biobank	DV	196,895	255,738	452,633	77	0.788	46.307 (29.852–217.432)	3356 8662	https://personal.broadinstitute.org/ryank/Saxena_fullUKBB_Daytimenapping_summary_stats.zip
Daytime sleepiness	UK Biobank	DV	104,786	347,285	452,071	25	0.233	42.118 (30.105–117.438)	3140 9809	https://personal.broadinstitute.org/ryank/Saxena.fullUKBB.DaytimeSleepiness.sumstats.zip
Insomnia	UK Biobank	DV	108,357	345,022	453,379	28	0.265	42.84 (29.241–181.158)	3080 4566	https://personal.broadinstitute.org/ryank/Saxena_fullUKBB_Insomnia_summary_stats.zip
Long sleep duration	UK Biobank	DV	34,184	305,742	339,926	6	0.055	41.135 (29.889–52.982)	3084 6698	https://personal.broadinstitute.org/ryank/longsumstats.txt.zip
Morning person	UK Biobank	DV	252,287	150,908	403,195	99	1.099	44.758 (29.019–168.521)	3069 6823	https://personal.broadinstitute.org/ryank/morning_person_BOLT.output_HRC.only_plus.metrics_maf0.001_hwep1em12_info0.3_logORs.txt.gz
Short sleep duration	UK Biobank	DV	106,192	305,742	411,934	16	0.137	38.324 (29.901–77.041)	3084 6698	https://personal.broadinstitute.org/ryank/shortsumstats.txt.zip
Obstructive sleep apnea	FinnGen	DV	16,761	201,194	217,955	12	0.110	34.478 (30.085–42.202)	3324 3845	https://gwas.mrcieu.ac.uk/datasets/finn-b-G6 _SLEEPAPNO/
Sleep duration	UK Biobank	CV	446,118	–	446,118	47	0.429	40.734 (30.214–220.873)	3084 6698	https://personal.broadinstitute.org/ryank/sleepdurationsumstats.txt.zip
Snoring	UK Biobank	DV	151,836	255,230	407,066	20	0.204	41.497 (29.898–74.508)	3206 0260	https://gwas.mrcieu.ac.uk/files/ebi-a-GCST009761/ebi-a-GCST009761.vcf.gz
PDR	FinnGen	DV	9,511	362,581	372,092	6	0.122	75.672 (38.742–175.766)	3665 3562	https://storage.googleapis.com/finngen-public-data-r9/summary_stats/finngen_R9_DM_RETINA_PROLIF.gz

PDR, proliferative diabetic retinopathy; CV, continuity variables; DV, dichotomous variable; VEP, variance explained; PMID, Pubmed ID.

### Instrumental variable selection

Single-nucleotide polymorphisms (SNPs) associated with sleep phenotypes were selected as instrumental variables at the genome-wide significance threshold (*P* < 5 × 10^−8^). To mitigate against co-linearity between SNPs, linkage disequilibrium (LD) analysis was performed to select independent SNPs with r^2^ < 0.001 and the clumping window of 10, 000 kb. Besides, proxy SNPs were also excluded from follow-up analysis. To avoid the potential effects of weak instrument bias, the strength of the genetic instrument was evaluated by F-statistics, and variance was explained (R^2^)^25,26^. The values of F statistic > 10 were considered robust MR instruments. Moreover, PhenoScanner (http://www.phenoscanner.medschl.cam.ac.uk/) was used to evaluate the association of instrumental variables with confounding or risk factors for outcomes. The statistical power calculations for the MR analysis was performed using an online tool at http://cnsgenomics.com/shiny/mRnd/^27^.

### Data for outcome

GWAS summary data on PDR from FinnGen comprised 9511 cases and 362 581 controls (Table 1)^22^. The FinnGen study is a large personalized medicine project covering 500,000 Finnish biobank participants, and aims to provide the evidence of genomic effect on human health. The overall statistical analysis of the FinnGen study have already been published^22^. PDR, characterized by the progression of retinal neovascularization, is defined as the most advanced stage of diabetic eye disease in ICD-10 (code: H36.03*). To further assess the robustness of PDR GWAS, the PDR GWAS data were re-analyzed in our study. More detailed demographic characteristics of the participants of the PDR GWAS data can be obtained at https://r9.risteys.finngen.fi/endpoints/DM_RETINA_PROLIF#dialog-view-original-rules.

### Validation cohorts

To validate the reliability of the MR results in the FinnGen cohort, the GWAS data of insomnia and sleep duration in the UK Biobank subjects were retrieved from the IEU-OpenGWAS project (https://gwas.mrcieu.ac.uk/datasets/ukb-b-4424/, and https://gwas.mrcieu.ac.uk/datasets/ukb-b-3957/ ).

### Statistical analysis

A two-sample MR method was employed to evaluate the causal effects of sleep phenotypes on PDR. Results are presented as odds ratios (ORs) and 95% confidence intervals (CIs). IVW MR analysis with a random‐effects model was applied as the primary MR analysis to estimate the causal association between the exposure and the outcome^28^. However, the IVW method is sensitive to invalid instrumental variables and pleiotropy^29^. Moreover, MR Egger regression, weighted median, simple mode, and weighted mode methods were employed as complementary analysis to test the consistency of causal estimates. The weighted median method provides consistent estimates, although half of the genetic variants are invalid instrumental variables^29^. The MR-Egger regression was applied to detect and adjust for pleiotropy, but the statistical power was low^30^. Bonferroni correction was used to adjust for multiple tests involving 10 sleep phenotypes. Statistical significance was defined as *P* < 0.005 (significance level 0.05/10 MR tests) for all analysis.

### Sensitivity analysis

To assess the potential violation of the MR assumptions, a series of MR sensitivity analysis was performed to test the stability and reliability of the results. Cochran Q test and the I^2^ statistic were used to evaluate the heterogeneity. A fixed-effects model was used in case of significant heterogeneity; otherwise, a random-effects model was used^31^. The MR-Egger regression method and the intercept test were performed to evaluate horizontal pleiotropy. Besides, the MR pleiotropy residual sum and outlier (MR-PRESSO) method was also applied to test for possible bias from horizontal pleiotropy and outlier variants removal^32^. A leave-one-out analysis was utilized for further sensitivity analysis. The MR Steiger test of directionality was performed to test the causal direction between sleep phenotypes and the PDR outcomes and remove SNPs that have a more significant association with the outcome than sleep phenotypes^33^.

### Linkage disequilibrium score regression (LDSC)

LDSC was used to estimate the genetic correlations between sleep phenotypes and PDR outcomes^34^.

### Colocalization analysis

Colocalization analysis, a Bayesian-based method, provides five posterior probabilities for these hypotheses (PPH_0_: no causal variants for either trait; PPH_1_: a causal variant for trait 1; PPH_2_: a causal variant for trait 2; PPH_3_: two different causal variants for trait 1 and trait 2; and PPH_4_: a shared causal variant between two traits)^35^. Colocalization analysis was performed to examine whether sleep phenotypes and PDR share a common causal variant in a given region. For each of these sleep phenotypes and PDR pairs, the genomic region extending 1000 kb on both sides of the lead sleep phenotypes variant was used, and PPH_4_ > 75% was considered to have strong colocalization evidence.

### Reverse MR analysis

To further investigate whether there is genetic evidence for a reverse causal effect of PDR on sleep phenotypes, a bi-directional MR analysis was performed with PDR as exposure and sleep phenotypes as outcomes.

### Statistical analysis and analysis software

This MR study followed the guidelines for strengthening the reporting of observational studies in epidemiology–MR (STROBE-MR)^36^. Statistical analyses were performed in R (version 4.1.2) Software, and MR analysis was performed using the TwoSampleMR package, and MR-PRESSO analysis was performed using the MRPRESSO package^32,37^. Colocalization analysis was used the Coloc package^35^.

### Ethical approval and consent to participate

All participating studies of GWAS have obtained approval from relevant institutional review boards, and written informed consent was received from all subjects. Summary-level data in our study are publicly available. The Medical Ethics Committee of The First Affiliated Hospital of Henan University of Science and Technology ruled that no formal ethics approval was required for this study.

## Results

### Genetic instruments

In the present study, 122/77/25/28/6/99/16/12/47/20 SNPs were selected as genetic instruments for sleep phenotypes (Chronotype/Daytime napping/Daytime sleepiness/Insomnia/Long sleep duration/Morning person/Short sleep duration/Obstructive sleep apnea/Sleep duration/Snoring). The F statistics for all genetic instruments were > 10 (Table 1). Summary statistics data for the SNPs–exposure associations are presented in Supplementary Table 1. The six locus polymorphisms are significantly associated with PDR (*P* < 5 × 10^−8^) (Supplementary Table 2). Manhattan and QQ plots are shown in Supplementary Fig. 1A and B. Power analysis indicated all MR analysis were sufficiently powered (Supplementary Table 3).

### MR analysis of sleep phenotypes on PDR risks

**Figure 2:**
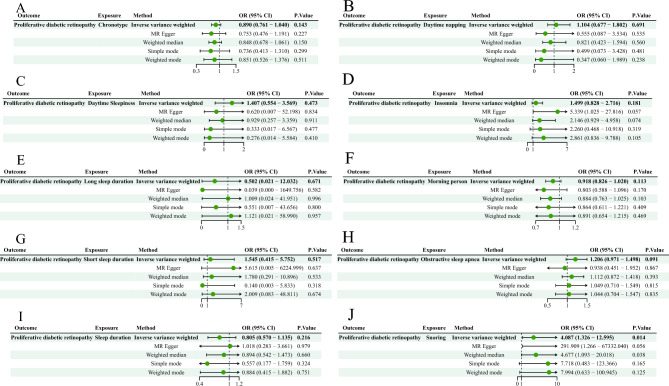
The causal effect of genetically predicted sleep phenotypes on the risk of PDR.

Two-sample MR analysis was initially performed to evaluate the causal effect of sleep phenotypes on PDR. Based on the IVW analysis results before Bonferroni correction, genetic predisposition to snoring was associated with increased risk of PDR (OR = 4.087 (95% CI 1.326–12.595), *P* = 0.014). In contrast, genetically predicted chronotype(OR = 0.890, 95% CI 0.761–1.040, *P* = 0.143), daytime napping (OR = 1.104, 95% CI 0.677–1.802, *P* = 0.691), daytime sleepiness (OR = 1.407, 95% CI 0.554–3.569, *P* = 0.473), insomnia (OR = 1.499, 95% CI 0.828–2.716, *P* = 0.181), long sleep duration (OR = 0.502, 95% CI 0.021–12.032, *P* = 0.671), morning person (OR = 0.918, 95% CI 0.826–1.020, *P* = 0.113), short sleep duration (OR = 1.545, 95% CI 0.415–5.752, *P* = 0.517), obstructive sleep apnea (OR = 1.206, 95% CI 0.971–1.498, *P* = 0.091), sleep duration (OR = 0.805, 95% CI 0.570–1.13, *P* = 0.216) were not causally associated with PDR risk. However, no causal relationship existed between genetically predicted sleep phenotypes and PDR after Bonferroni correction (Fig. 2).

### Sensitivity analysis

According to Cochran’s Q test of heterogeneity using the IVW method, there was evidence of heterogeneity among individual SNP effect estimates in daytime napping on PDR (Q = 107.023, I^2^ = 29%, *P* = 0.011, Table 2). Therefore, a fixed-effects IVW model would be implemented. Similarly, there was no causal association between genetically predicted daytime napping and PDR (OR = 1.104, 95% CI 0.731–1.668, *P* = 0.638). Moreover, no evidence for horizontal pleiotropy was observed using the MR-Egger regression method (*P* > 0.05, Table 2). Additionally, the MR-PRESSO outlier test detected potential outliers, and re-analysis was carried out after removing outliers from genetic instruments. Furthermore, a leave-one-out analysis was also performed, and the results are presented in Supplementary Fig. 2.

**Table 2:** Heterogeneity and horizontal pleiotropy analyses between sleep phenotypes and PDR.

Exposure	Heterogeneity			Horizontal pleiotropy			MR PRESSO global test *P* value
	Q	*P* value	I2	Intercept	Se	*P* value	
Chronotype	146.419	0.058	0.174	0.004	0.005	0.449	0.059
Daytime napping	107.023	0.011	0.290	0.007	0.009	0.452	0.014
Daytime Sleepiness	25.709	0.368	0.066	0.006	0.017	0.714	0.345
Insomnia	24.956	0.577	− 0.082	− 0.014	0.009	0.118	0.561
Long sleep duration	5.049	0.410	0.010	0.017	0.035	0.644	0.530
Morning person	117.045	0.092	0.163	0.005	0.006	0.371	0.095
Short sleep duration	16.126	0.374	0.070	− 0.009	0.024	0.719	0.376
Obstructive sleep apnea	15.314	0.169	0.282	0.016	0.023	0.497	0.186
Sleep duration	60.779	0.071	0.243	− 0.004	0.011	0.710	0.076
Snoring	25.424	0.147	0.253	− 0.033	0.021	0.134	0.177

Q, Cochran’s Q value.

### Direction validation and confounders

Steiger direction test was performed to examine whether there was reverse causality between sleep phenotypes and PDR, where the results did not support the existence of reverse causal effects between the two. Some SNPs were associated with the known confounders (body mass index, glucose, diabetes, alcohol, smoking, cholesterol, triglycerides, high-density lipoprotein, low-density lipoprotein, blood pressure, insulin, and obesity), which were excluded from further analysis.

### Results of genetic correlation analysis

The LDSC analysis results do not indicate any evidence for a genetic correlation between sleep phenotypes and PDR (Chronotype: r_g_ = 0.0132, se = 0.0538, *P* = 0.8068; Daytime napping: r_g_ = 0.0460, se = 0.0568, *P* = 0.4172; Daytime sleepiness: r_g_ = 0.1022, se = 0.0601, *P* = 0.089; Insomnia: r_g_ = 0.0789, se = 0.0555, *P* = 0.1551; Long sleep duration: r_g_ = − 0.0003, se = 0.0817, *P* = 0.9971; Morning person: r_g_ = 0.0115, se = 0.0564, *P* = 0.8390; Short sleep duration: r_g_ = 0.0905, se = 0.0637, *P* = 0.1557; Obstructive sleep apnea: r_g_ = 0.1387, se = 0.0697, *P* = 0.0465; Sleep duration: r_g_ = − 0.0578, se = 0.0629, *P* = 0.3576; Snoring: r_g_ = 0.0482, se = 0.0578, *P* = 0.4045) (Table 3).

**Table 3:** Genetic correlations between sleep phenotypes and PDR.

Exposure	Outcome	rg	rg_se	rg_p
Chronotype	Proliferative diabetic retinopathy	0.013	0.054	0.807
Daytime napping	Proliferative diabetic retinopathy	0.046	0.057	0.417
Daytime sleepiness	Proliferative diabetic retinopathy	0.102	0.060	0.089
Insomnia	Proliferative diabetic retinopathy	0.079	0.056	0.155
Long sleep	Proliferative diabetic retinopathy	0.000	0.082	0.997
Morning person	Proliferative diabetic retinopathy	0.012	0.056	0.839
Short sleep	Proliferative diabetic retinopathy	0.091	0.064	0.156
Sleep apnoea	Proliferative diabetic retinopathy	0.139	0.070	0.047
Sleep duration	Proliferative diabetic retinopathy	− 0.058	0.063	0.358
Snoring	Proliferative diabetic retinopathy	0.048	0.058	0.405

### Results of genetic colocalization analysis

**Figure 3:**
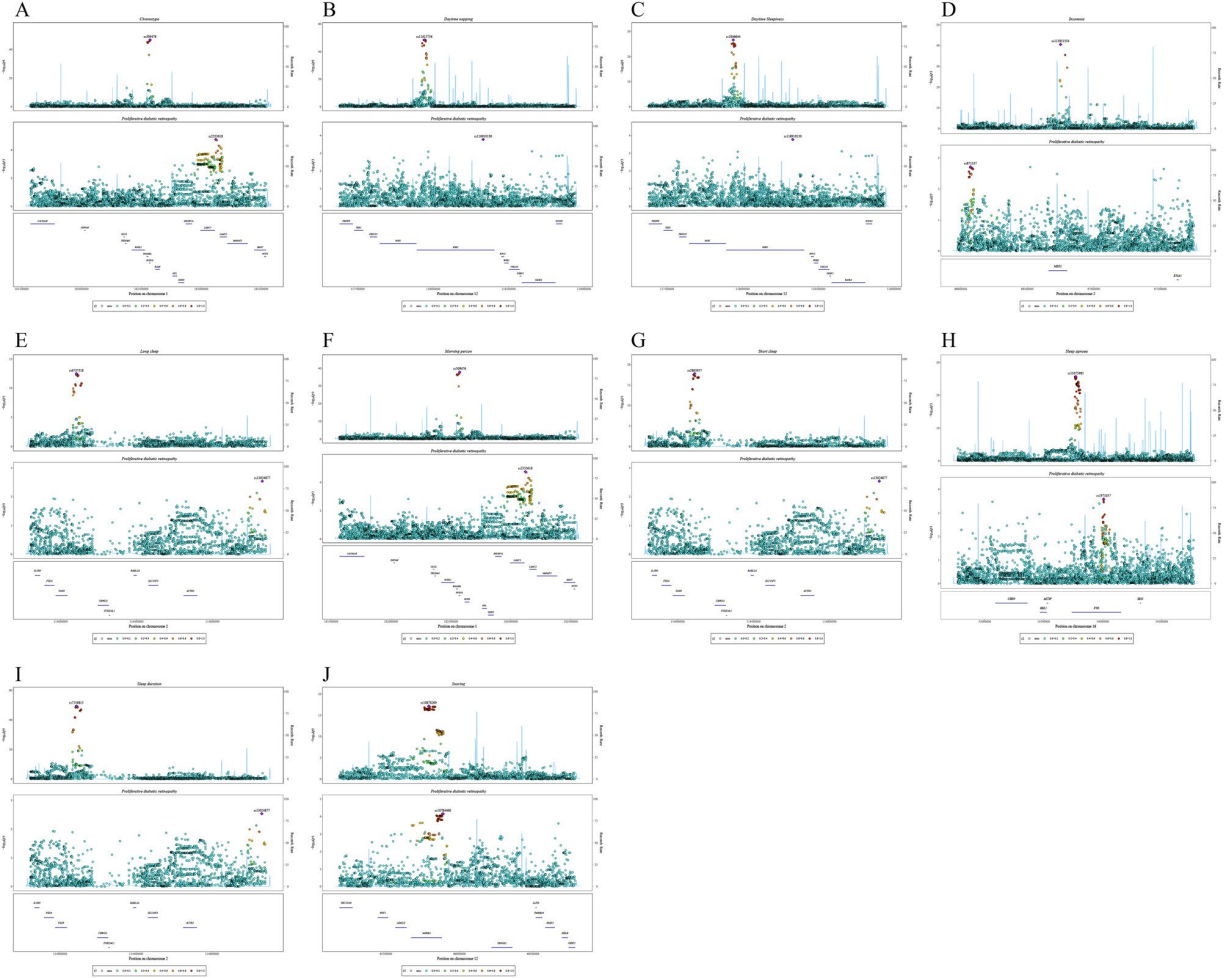
Genetic colocalization analysis between sleep phenotypes and PDR.

The colocalization analysis results suggested that sleep phenotypes and PDR were unlikely to share a causal variant within the same locus. (Chronotype: PPH_4_ = 2%; Daytime napping: PPH_4_ = 4%; Daytime Sleepiness: PPH_4_ = 3%; Insomnia: PPH_4_ = 4%; Long sleep duration: PPH_4_ = 1%; Morning person: PPH_4_ = 2%; Short sleep duration: PPH_4_ = 1%; Obstructive sleep apnea: PPH_4_ = 1%; Sleep duration: PPH_4_ = 1%; Snoring: PPH_4_ = 40%) (Table 4 and Fig. 3).

**Table 4:** Genetic colocalization analysis between sleep phenotypes and PDR.

Exposure	PP.H0.abf	PP.H1.abf	PP.H2.abf	PP.H3.abf	PP.H4.abf
Chronotype	1.78E-41	0.39	2.67E-41	0.59	0.02
Daytime napping	1.14E-42	0.72	3.91E-43	0.25	0.04
Daytime sleepiness	3.72E-20	0.71	1.37E-20	0.26	0.03
Insomnia	2.16E-34	0.71	7.62E-35	0.25	0.04
Long sleep duration	1.02E-06	0.83	1.95E-07	0.16	0.01
Morning person	9.65E-33	0.39	1.45E-32	0.59	0.02
Short sleep duration	6.82E-12	0.84	1.29E-12	0.16	0.01
Obstructive sleep apnea	3.74E-21	0.57	2.81E-21	0.43	0.01
Sleep duration	1.65E-43	0.83	3.12E-44	0.16	0.01
Snoring	2.45E-12	0.28	2.78E-12	0.32	0.40

### Results of MR analysis in the UK subjects

We replicated the MR analysis on the UK Biobank cohort. Genetically predicted insomnia (OR = 1.304, 95% CI 0.810–2.099, *P* = 0.275), sleep duration (OR = 0.846, 95% CI 0.581–1.231, *P* = 0.382) were not causally associated with PDR risk based on IVW analysis (Supplementary Table 4).

### Results of reverse MR analysis

**Figure 4:**
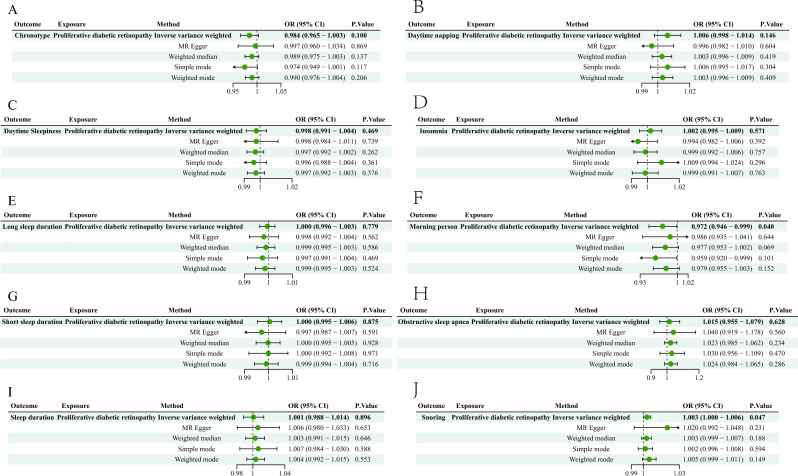
The causal effect of genetically predicted PDR on the risk of sleep phenotypes.

The results of an inverse MR analysis showed no causal association between PDR and sleep phenotypes (Fig. 4).

## Discussion

The current study explored the bidirectional causal association between sleep phenotypes and PDR, and SNP-based genetic correlation was evaluated. Results suggested no evidence of causal associations of daytime napping, daytime sleepiness, chronotype, morning person, insomnia, sleep duration, short, and long sleep duration, obstructive sleep apnea, snoring, and PDR. Additionally, the sensitivity analysis revealed cemented the robustness and reliability of the results. The genetic correlation analysis also did not provide strong evidence supporting a causal association between sleep phenotypes and PDR, further supported by the lack of sharing a common causal variant in colocalization analysis results. This is the first study to evaluate the effects of genetically predicted sleep phenotypes and PDR using MR analysis.

Moreover, this study did not provide any evidence regarding genetically predicted sleep phenotypes playing a role in the increased PDR risk, which was contrary to several previous observational studies. Tan et al.’ study reported that short and long sleep duration increased the risk of DR compared to normal sleep duration^38^. Similarly, a longitudinal study on 230 recruited diabetes type 2 patients found that obstructive sleep apnea was an independent risk factor for DR^39^. Besides, a retrospective observational study also identified severe obstructive sleep apnea associated with a higher prevalence of DR, proliferative DR, and diabetic macular edema (DME)^40^. Daytime sleepiness was also reported to be associated with vision-threatening DR, and insomnia as well to be associated with increased susceptibility to DR, vision-threatening DR, and DME^38,41^. In addition, An observational study found that OSA diagnosed by questionnaires and diagnosis codes was not significantly associated with DR, while OSA diagnosed by objective sleep assessments was significantly associated with DR^42^.

However, several controversial results also have been reported by relevant studies. Raman et al. confirmed that short and long sleep duration are not risk factors for DR in women and men^43^. The prospective case–control study and meta-analysis results also suggested that obstructive sleep apnea was not associated with DR^44,45^.

The effect of snoring, daytime napping, chronotype, and morning person on PDR has been negligibly studied. The novelty of our work lies for the first time in reporting no causal effects of these sleep phenotypes on PDR.

Furthermore, our results also conflict with previous observational studies regarding the relationship between sleep phenotypes and PDR. However, most previous studies were prospective or retrospective cohorts and cross‐sectional studies, given the nature of the observational, selection bias and unmeasured confounders cannot be excluded and causality cannot be determined. Mendelian randomization could reduce the influence of unknown or unmeasured confounders. Confounding factors may explain the findings between the present study and previous observational studies. For example, the role of the gut-retina" axis in DR has increasingly been recognized^46^. Sleep disorders can affect the composition of the intestinal microbiota^47^. A recent MR study has demonstrated that gut microbiota positively affects DR^48^. Sleep phenotypes might result in DR by modulating the intestinal microbiota^49^. In addition, inflammation may be a potential confounder factor for previous observational studies since it plays a fundamental role in DR pathogenesis by disrupting the retinal blood barrier^50^. Sleep phenotypes might indirectly participate in the prevalence of DR by modulating insulin sensitivity and affecting blood glucose levels^51^. In addition, Sleep rhythm disorders could lead to chronic metabolic disorders of melatonin. The melatonin levels of the DR group were significantly lower than those of the no-DR group^42^. Melatonin could significantly reduce the inflammatory markers levels of tumor necrosis factor-α, interleukin-1β, and inducible nitric oxide synthase (iNOS) in DR^52^. Sleep phenotypes may indirectly affect the occurrence and development of DR by regulating the level of melatonin. Our results further emphasize the need to explore the potential causal association between sleep phenotypes and PDR.

Our results were robust and reliable, along with detecting heterogeneity and horizontal pleiotropy results via sensitivity analysis. In addition, leave-one-out analysis was used to validate the effect of a single SNP on the causal relationship between sleep phenotypes and PDR. MR-PRESSO method was performed to recognize and remove outlying SNPs that might cause horizontal pleiotropy effects. The sample size was sufficiently large to provide enough power for the statistical analysis in this study. Our results were unlikely to suffer weak instrument bias because the F-statistics for the instrumental variables were > 10. Moreover, the PhenoScanner database was also used to detect potential pleiotropic SNPs. Body mass index, glucose, diabetes, alcohol, smoking, cholesterol, triglycerides, high-density lipoprotein, low-density lipoprotein, blood pressure, insulin, and obesity were considered as potential confounders in this study^53,54^.

This study had several strengths. First, gene variants were used as instrumental variables to infer causal relationships between sleep phenotypes and PDR, which reduced the effect of confounders and reverse causation. Second, the impact of snoring, daytime napping, chronotype, and morning person on PDR risk was reported for the first time in this stud. Third, SNPs as instrumental variables were derived from large-scale GWASs, providing reliable estimates for the causal relationships between sleep phenotypes and PDR and less vulnerability to weak instrumental bias. We further validated our results using genetic correlation analysis and colocalization analysis.

However, our study also had several limitations. First, Sleep phenotypes in different human populations may have different genetic underpinnings. In a recent genome-wide association study (GWAS) meta-analysis for sleep duration, Takeshi et al.^55^ reported that PAX8 and VRK2 gene polymorphisms were not associated with sleep duration in Japanese individuals but were associated with sleep duration in the UK population. Our study was mainly based on Europeans, indicating that results may not be generalizable to other ethnic groups, necessitating validation in different populations. Second, most of the GWAS data on the sleep phenotypes were derived from self-reported questionnaire results. This may lead to exposure misclassification and potential bias. Thus, further prospective sleep evaluation using objective sleep parameters is warranted to understand better the relationship between sleep phenotypes and the risk of PDR. Finally. Although the samples of exposures and outcomes are from different cohorts, there is a potential overlap between exposures and outcomes, leading to the possibility of results bias.

## Conclusion

Our study did not support the causal effect between sleep phenotypes and PDR. Further longitudinal studies are warranted to validate the findings. In addition, the impact effect of sleep phenotypes diagnosed by clinicians and PDR needs to be investigated in the future.

### Supplementary Information

Supplementary Information 1.

Supplementary Information 2.

Supplementary Information 3.

Supplementary Information 4.

Supplementary Information 5.

Supplementary Information 6.

## Data Availability

All data generated or analyzed during this study are included in this published article. The raw data can be obtained from the IEU Open GWAS database (https://gwas.mrcieu.ac.uk/) and the Sleep Disorder Knowledge Portal project (https://sleep.hugeamp.org/datasets.html).

## Abbreviations

SNPs: Single nucleotide polymorphisms
DR: Diabetic retinopathy
PDR: Proliferative diabetic retinopathy
IVW: Inverse‐variance weighted
MR: Mendelian randomization
OSA: Obstructive sleep apnea
GWAS: Genome-wide association studies
IEU: Integrative epidemiology unit
LD: Linkage disequilibrium
ORs: Odds ratios
LDSC: Linkage disequilibrium score regression
DME: Diabetic macular edema
