# Supplementary figures and images for: No bidirectional relationship between sleep phenotypes and risk of proliferative diabetic retinopathy: a two-sample Mendelian randomization study
Source: Sci Rep. 2024 Apr 26;14:9585. doi: 10.1038/s41598-024-60446-3 (PMC11053118; doi:10.1038/s41598-024-60446-3)

A

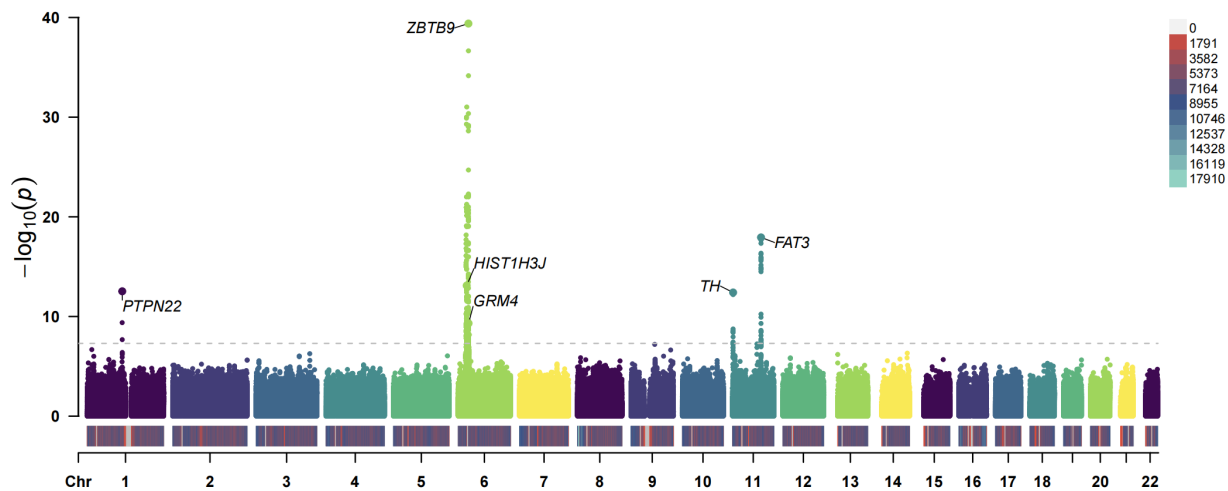

B

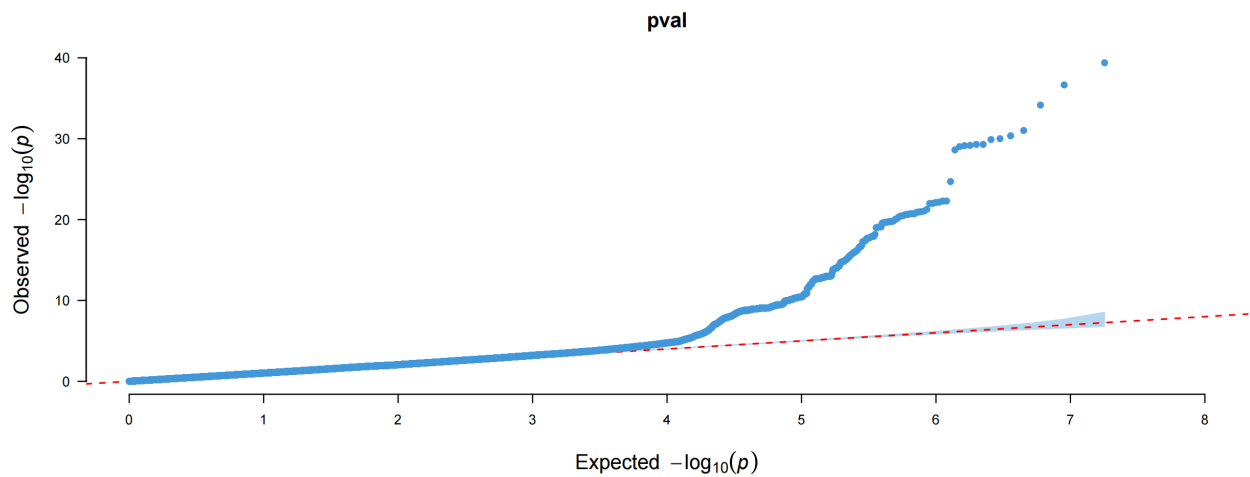

Supplement: Supplementary file 1 — Supplementary Information 1. [file 41598_2024_60446_MOESM1_ESM.pdf]

A

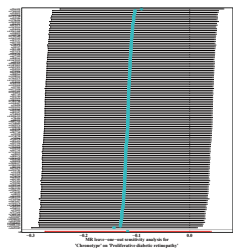

B

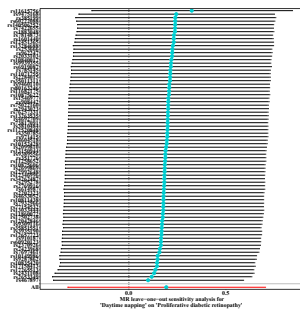

C

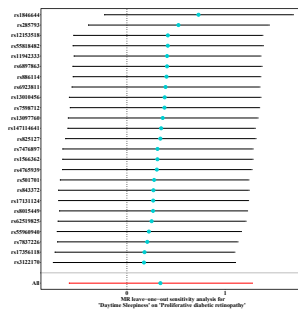

D

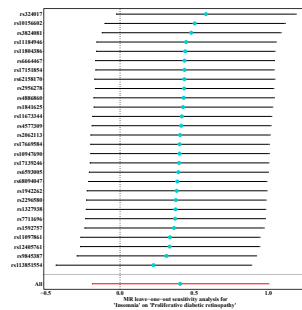

E

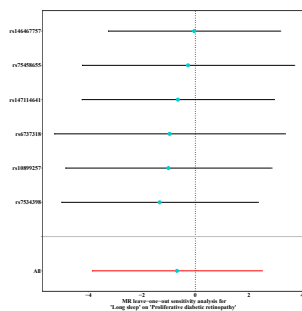

F

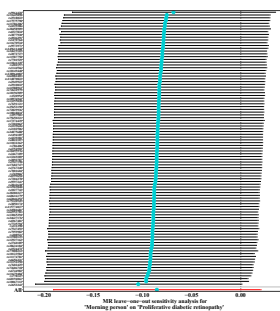

G

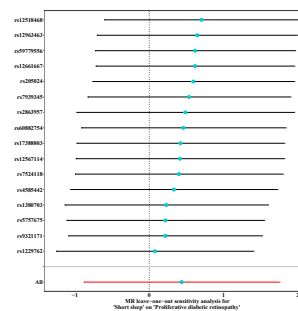

H

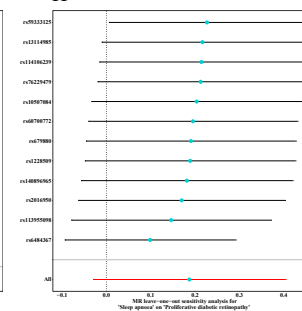

I

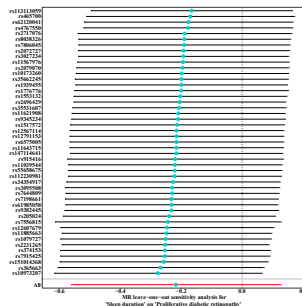

J

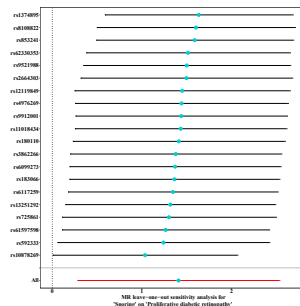

Supplement: Supplementary file 2 — Supplementary Information 2. [file 41598_2024_60446_MOESM2_ESM.pdf]
