# Supplementary material for: No bidirectional relationship between sleep phenotypes and risk of proliferative diabetic retinopathy: a two-sample Mendelian randomization study
Source: Sci Rep. 2024 Apr 26;14:9585. doi: 10.1038/s41598-024-60446-3 (PMC11053118; doi:10.1038/s41598-024-60446-3)
Supplement: Supplementary file 3 — Supplementary Information 3. [file 41598_2024_60446_MOESM3_ESM.docx]

**Supplementary table 1.** SNPs are used as genetic instrumental variables for sleep phenotypes.

|  |  | Exposure | | | | | |  | Outcome | | |
| --- | --- | --- | --- | --- | --- | --- | --- | --- | --- | --- | --- |
| exposure | SNP | EA | OA | EAF | se | beta | p |  | se | beta | p |
| Chronotype | rs10067113 | C | T | 0.381 | 0.003 | -0.016 | 6.30E-09 |  | 0.016 | -0.036 | 0.021 |
| Chronotype | rs10149448 | A | G | 0.604 | 0.003 | 0.016 | 2.70E-09 |  | 0.015 | -0.017 | 0.263 |
| Chronotype | rs1027742 | A | G | 0.738 | 0.003 | 0.018 | 2.80E-09 |  | 0.016 | 0.007 | 0.678 |
| Chronotype | rs10280205 | T | C | 0.692 | 0.003 | 0.017 | 1.40E-09 |  | 0.015 | -0.002 | 0.872 |
| Chronotype | rs10402849 | C | T | 0.799 | 0.003 | -0.019 | 8.90E-09 |  | 0.016 | 0.036 | 0.030 |
| Chronotype | rs10495976 | A | T | 0.610 | 0.003 | -0.018 | 5.10E-11 |  | 0.015 | 0.003 | 0.853 |
| Chronotype | rs10823233 | T | C | 0.452 | 0.003 | -0.015 | 4.30E-08 |  | 0.015 | -0.019 | 0.204 |
| Chronotype | rs10988239 | C | T | 0.487 | 0.003 | 0.019 | 1.60E-11 |  | 0.015 | 0.023 | 0.119 |
| Chronotype | rs11032362 | G | A | 0.909 | 0.005 | -0.040 | 4.80E-17 |  | 0.032 | 0.009 | 0.777 |
| Chronotype | rs1113295 | T | G | 0.441 | 0.003 | 0.016 | 6.80E-09 |  | 0.015 | 0.017 | 0.245 |
| Chronotype | rs11227452 | G | A | 0.828 | 0.004 | -0.019 | 4.60E-08 |  | 0.019 | 0.010 | 0.615 |
| Chronotype | rs114352914 | G | A | 0.812 | 0.003 | -0.020 | 7.90E-09 |  | 0.018 | 0.012 | 0.493 |
| Chronotype | rs114670539 | C | T | 0.942 | 0.006 | -0.034 | 4.60E-09 |  | 0.045 | 0.058 | 0.193 |
| Chronotype | rs114848860 | A | T | 0.976 | 0.009 | -0.056 | 3.60E-10 |  | 0.046 | -0.051 | 0.264 |
| Chronotype | rs114870822 | G | A | 0.987 | 0.012 | -0.070 | 2.00E-09 |  | 0.067 | 0.063 | 0.343 |
| Chronotype | rs11587758 | G | A | 0.604 | 0.003 | -0.024 | 5.50E-19 |  | 0.015 | 0.017 | 0.247 |
| Chronotype | rs11588913 | G | A | 0.602 | 0.003 | 0.015 | 1.50E-08 |  | 0.015 | -0.012 | 0.441 |
| Chronotype | rs11670534 | C | T | 0.834 | 0.004 | 0.021 | 8.50E-09 |  | 0.022 | -0.043 | 0.049 |
| Chronotype | rs11748798 | G | T | 0.750 | 0.003 | 0.020 | 5.20E-11 |  | 0.017 | 0.000 | 0.991 |
| Chronotype | rs11786306 | G | C | 0.646 | 0.003 | -0.018 | 8.70E-11 |  | 0.015 | 0.029 | 0.057 |
| Chronotype | rs11924337 | G | C | 0.728 | 0.003 | -0.019 | 2.20E-10 |  | 0.020 | -0.050 | 0.010 |
| Chronotype | rs12432176 | C | A | 0.621 | 0.003 | -0.016 | 6.50E-09 |  | 0.015 | -0.019 | 0.199 |
| Chronotype | rs12541362 | A | T | 0.650 | 0.003 | -0.021 | 3.50E-14 |  | 0.016 | -0.004 | 0.809 |
| Chronotype | rs12811046 | A | G | 0.553 | 0.003 | 0.017 | 6.20E-10 |  | 0.015 | -0.005 | 0.717 |
| Chronotype | rs12927162 | A | G | 0.722 | 0.003 | 0.029 | 6.80E-22 |  | 0.016 | 0.006 | 0.710 |
| Chronotype | rs12969848 | C | T | 0.470 | 0.003 | -0.022 | 1.30E-15 |  | 0.015 | -0.003 | 0.836 |
| Chronotype | rs13065394 | G | T | 0.712 | 0.003 | 0.018 | 3.00E-09 |  | 0.016 | 0.010 | 0.530 |
| Chronotype | rs13255030 | A | G | 0.585 | 0.003 | 0.016 | 2.80E-09 |  | 0.015 | -0.010 | 0.531 |
| Chronotype | rs13316611 | G | T | 0.743 | 0.003 | -0.018 | 1.70E-08 |  | 0.017 | 0.008 | 0.635 |
| Chronotype | rs140206235 | C | T | 0.981 | 0.010 | -0.055 | 2.30E-08 |  | 0.102 | 0.033 | 0.749 |
| Chronotype | rs149611468 | T | C | 0.988 | 0.013 | 0.078 | 8.50E-10 |  | 0.050 | -0.062 | 0.214 |
| Chronotype | rs17267683 | C | G | 0.761 | 0.003 | 0.020 | 2.70E-10 |  | 0.017 | -0.026 | 0.139 |
| Chronotype | rs17448682 | C | T | 0.768 | 0.003 | -0.022 | 2.20E-12 |  | 0.017 | 0.004 | 0.840 |
| Chronotype | rs17575798 | G | A | 0.807 | 0.003 | 0.023 | 4.20E-11 |  | 0.018 | -0.036 | 0.053 |
| Chronotype | rs17604349 | G | A | 0.821 | 0.004 | 0.026 | 1.70E-13 |  | 0.017 | -0.034 | 0.051 |
| Chronotype | rs1800828 | C | G | 0.746 | 0.003 | 0.018 | 1.70E-08 |  | 0.018 | 0.012 | 0.502 |
| Chronotype | rs1947198 | C | T | 0.878 | 0.004 | -0.024 | 1.80E-09 |  | 0.020 | 0.003 | 0.891 |
| Chronotype | rs1952923 | G | A | 0.261 | 0.003 | 0.017 | 3.60E-08 |  | 0.017 | 0.009 | 0.622 |
| Chronotype | rs197273 | A | G | 0.470 | 0.003 | 0.016 | 1.30E-08 |  | 0.015 | -0.014 | 0.338 |
| Chronotype | rs202157 | C | T | 0.299 | 0.003 | 0.026 | 4.10E-18 |  | 0.018 | -0.019 | 0.304 |
| Chronotype | rs2072727 | T | C | 0.436 | 0.003 | 0.016 | 8.50E-10 |  | 0.015 | -0.021 | 0.166 |
| Chronotype | rs2239626 | T | C | 0.695 | 0.003 | -0.020 | 2.30E-12 |  | 0.018 | 0.007 | 0.697 |
| Chronotype | rs226090 | C | T | 0.168 | 0.004 | 0.021 | 4.10E-09 |  | 0.020 | -0.007 | 0.723 |
| Chronotype | rs2311837 | T | C | 0.592 | 0.003 | -0.015 | 3.60E-08 |  | 0.015 | -0.028 | 0.057 |
| Chronotype | rs231398 | G | A | 0.838 | 0.004 | 0.021 | 8.40E-09 |  | 0.019 | 0.013 | 0.483 |
| Chronotype | rs2422413 | T | C | 0.622 | 0.003 | -0.016 | 3.60E-09 |  | 0.016 | -0.029 | 0.063 |
| Chronotype | rs2518022 | T | C | 0.084 | 0.005 | 0.040 | 4.80E-17 |  | 0.031 | -0.064 | 0.037 |
| Chronotype | rs2653349 | A | G | 0.215 | 0.003 | 0.039 | 5.20E-32 |  | 0.019 | 0.038 | 0.042 |
| Chronotype | rs2712056 | C | T | 0.815 | 0.003 | -0.022 | 5.10E-11 |  | 0.022 | 0.019 | 0.377 |
| Chronotype | rs28380327 | A | T | 0.629 | 0.003 | 0.021 | 2.90E-13 |  | 0.016 | 0.007 | 0.658 |
| Chronotype | rs28458909 | C | T | 0.876 | 0.004 | 0.043 | 2.60E-26 |  | 0.019 | -0.022 | 0.244 |
| Chronotype | rs28634184 | C | T | 0.749 | 0.003 | 0.018 | 2.80E-08 |  | 0.018 | 0.019 | 0.285 |
| Chronotype | rs2881955 | C | T | 0.721 | 0.003 | -0.016 | 4.30E-08 |  | 0.017 | -0.013 | 0.437 |
| Chronotype | rs2949923 | A | G | 0.538 | 0.003 | -0.016 | 2.10E-09 |  | 0.015 | 0.013 | 0.386 |
| Chronotype | rs2968511 | C | G | 0.695 | 0.003 | -0.017 | 4.90E-09 |  | 0.018 | -0.029 | 0.111 |
| Chronotype | rs2971970 | T | G | 0.218 | 0.003 | 0.019 | 6.00E-09 |  | 0.020 | -0.027 | 0.173 |
| Chronotype | rs308521 | T | C | 0.603 | 0.003 | 0.020 | 3.70E-13 |  | 0.016 | 0.014 | 0.378 |
| Chronotype | rs3100052 | A | G | 0.387 | 0.003 | 0.017 | 6.90E-10 |  | 0.015 | -0.023 | 0.115 |
| Chronotype | rs34239319 | G | T | 0.898 | 0.004 | -0.028 | 3.90E-10 |  | 0.027 | -0.027 | 0.321 |
| Chronotype | rs34340407 | T | C | 0.753 | 0.003 | -0.018 | 4.30E-09 |  | 0.017 | 0.006 | 0.719 |
| Chronotype | rs34619169 | G | A | 0.692 | 0.003 | -0.017 | 3.80E-09 |  | 0.017 | 0.027 | 0.115 |
| Chronotype | rs35524253 | G | A | 0.643 | 0.003 | -0.016 | 5.80E-09 |  | 0.016 | 0.006 | 0.726 |
| Chronotype | rs359250 | G | T | 0.364 | 0.003 | -0.017 | 9.20E-10 |  | 0.016 | 0.000 | 0.982 |
| Chronotype | rs3760185 | C | T | 0.752 | 0.003 | 0.024 | 2.60E-14 |  | 0.019 | 0.016 | 0.403 |
| Chronotype | rs3808964 | G | T | 0.365 | 0.003 | -0.016 | 2.70E-08 |  | 0.015 | 0.012 | 0.419 |
| Chronotype | rs3816454 | T | G | 0.375 | 0.003 | 0.020 | 4.90E-13 |  | 0.015 | 0.001 | 0.946 |
| Chronotype | rs3850174 | T | A | 0.743 | 0.003 | 0.019 | 6.80E-10 |  | 0.018 | 0.010 | 0.576 |
| Chronotype | rs4141920 | G | A | 0.544 | 0.003 | 0.016 | 4.60E-09 |  | 0.015 | 0.031 | 0.036 |
| Chronotype | rs4237555 | C | T | 0.473 | 0.003 | -0.016 | 6.00E-10 |  | 0.015 | 0.021 | 0.171 |
| Chronotype | rs4239386 | T | A | 0.665 | 0.003 | 0.022 | 1.00E-14 |  | 0.015 | -0.025 | 0.101 |
| Chronotype | rs4246036 | C | T | 0.292 | 0.003 | 0.024 | 2.40E-15 |  | 0.016 | -0.022 | 0.172 |
| Chronotype | rs4339281 | A | G | 0.873 | 0.004 | 0.022 | 3.20E-08 |  | 0.022 | -0.006 | 0.777 |
| Chronotype | rs4714475 | G | T | 0.754 | 0.003 | 0.019 | 1.60E-09 |  | 0.016 | 0.009 | 0.565 |
| Chronotype | rs4822107 | G | A | 0.494 | 0.003 | -0.016 | 9.60E-09 |  | 0.015 | 0.004 | 0.779 |
| Chronotype | rs4838161 | T | G | 0.561 | 0.003 | 0.016 | 2.20E-09 |  | 0.015 | -0.015 | 0.321 |
| Chronotype | rs4936291 | A | G | 0.611 | 0.003 | -0.018 | 3.80E-10 |  | 0.015 | 0.026 | 0.085 |
| Chronotype | rs4948547 | C | A | 0.256 | 0.003 | 0.019 | 1.20E-09 |  | 0.017 | -0.001 | 0.938 |
| Chronotype | rs4949980 | A | G | 0.932 | 0.005 | -0.032 | 2.70E-09 |  | 0.020 | -0.006 | 0.781 |
| Chronotype | rs509476 | T | C | 0.030 | 0.008 | 0.114 | 5.70E-47 |  | 0.059 | -0.051 | 0.387 |
| Chronotype | rs56076457 | C | T | 0.471 | 0.003 | -0.016 | 1.30E-09 |  | 0.015 | -0.019 | 0.222 |
| Chronotype | rs56947091 | C | T | 0.489 | 0.003 | -0.019 | 6.00E-13 |  | 0.015 | 0.012 | 0.414 |
| Chronotype | rs57435966 | C | T | 0.913 | 0.005 | 0.055 | 2.60E-30 |  | 0.021 | 0.005 | 0.822 |
| Chronotype | rs59986227 | C | G | 0.742 | 0.003 | -0.018 | 8.30E-09 |  | 0.017 | -0.016 | 0.324 |
| Chronotype | rs60616179 | A | G | 0.945 | 0.006 | 0.038 | 1.10E-10 |  | 0.025 | 0.008 | 0.762 |
| Chronotype | rs6131942 | A | G | 0.420 | 0.003 | -0.018 | 9.40E-11 |  | 0.015 | -0.010 | 0.505 |
| Chronotype | rs61773390 | G | T | 0.804 | 0.003 | -0.034 | 1.20E-23 |  | 0.017 | 0.008 | 0.663 |
| Chronotype | rs62082401 | C | G | 0.809 | 0.003 | -0.026 | 1.60E-14 |  | 0.018 | 0.014 | 0.441 |
| Chronotype | rs62553781 | C | T | 0.965 | 0.007 | 0.050 | 1.20E-11 |  | 0.068 | 0.073 | 0.285 |
| Chronotype | rs6442446 | A | G | 0.291 | 0.003 | 0.017 | 3.10E-09 |  | 0.017 | -0.014 | 0.405 |
| Chronotype | rs6477309 | C | T | 0.335 | 0.003 | -0.017 | 8.00E-10 |  | 0.016 | 0.017 | 0.280 |
| Chronotype | rs6658041 | G | A | 0.400 | 0.003 | -0.015 | 1.70E-08 |  | 0.015 | -0.014 | 0.378 |
| Chronotype | rs6967481 | C | T | 0.503 | 0.003 | -0.021 | 6.90E-15 |  | 0.015 | -0.009 | 0.559 |
| Chronotype | rs698015 | C | T | 0.353 | 0.003 | -0.016 | 8.80E-09 |  | 0.016 | -0.009 | 0.584 |
| Chronotype | rs7148842 | C | T | 0.612 | 0.003 | 0.015 | 1.80E-08 |  | 0.015 | -0.004 | 0.780 |
| Chronotype | rs72632979 | A | G | 0.828 | 0.004 | 0.022 | 1.20E-09 |  | 0.029 | -0.028 | 0.341 |
| Chronotype | rs72829706 | A | G | 0.961 | 0.007 | 0.041 | 5.10E-09 |  | 0.032 | -0.011 | 0.730 |
| Chronotype | rs7304278 | A | G | 0.275 | 0.003 | -0.020 | 2.20E-11 |  | 0.017 | -0.001 | 0.939 |
| Chronotype | rs74357745 | A | G | 0.879 | 0.004 | 0.027 | 8.70E-11 |  | 0.023 | 0.038 | 0.103 |
| Chronotype | rs75120545 | C | T | 0.970 | 0.008 | -0.061 | 1.10E-13 |  | 0.036 | -0.007 | 0.848 |
| Chronotype | rs7547493 | A | G | 0.822 | 0.004 | -0.038 | 3.90E-27 |  | 0.017 | -0.003 | 0.839 |
| Chronotype | rs7602425 | C | T | 0.924 | 0.005 | -0.031 | 8.50E-10 |  | 0.038 | -0.072 | 0.055 |
| Chronotype | rs769066 | T | C | 0.817 | 0.003 | -0.022 | 3.20E-10 |  | 0.021 | -0.002 | 0.938 |
| Chronotype | rs7691121 | C | G | 0.768 | 0.003 | 0.022 | 5.60E-12 |  | 0.017 | -0.010 | 0.569 |
| Chronotype | rs7701529 | A | T | 0.238 | 0.003 | -0.019 | 3.60E-09 |  | 0.019 | 0.028 | 0.147 |
| Chronotype | rs7735794 | G | A | 0.776 | 0.003 | -0.020 | 4.70E-09 |  | 0.017 | -0.011 | 0.509 |
| Chronotype | rs77384811 | G | C | 0.843 | 0.004 | 0.025 | 2.80E-11 |  | 0.017 | 0.026 | 0.139 |
| Chronotype | rs786406 | A | G | 0.298 | 0.003 | -0.024 | 1.90E-16 |  | 0.015 | 0.004 | 0.795 |
| Chronotype | rs7959983 | T | C | 0.596 | 0.003 | -0.020 | 9.50E-13 |  | 0.016 | -0.011 | 0.473 |
| Chronotype | rs7977610 | A | T | 0.673 | 0.003 | 0.016 | 2.10E-08 |  | 0.016 | 0.032 | 0.051 |
| Chronotype | rs80097534 | G | T | 0.901 | 0.005 | 0.028 | 9.30E-10 |  | 0.022 | -0.005 | 0.838 |
| Chronotype | rs827749 | T | C | 0.672 | 0.003 | -0.024 | 1.00E-16 |  | 0.017 | 0.007 | 0.684 |
| Chronotype | rs885255 | T | C | 0.348 | 0.003 | 0.017 | 4.40E-09 |  | 0.016 | -0.004 | 0.817 |
| Chronotype | rs9348050 | T | C | 0.489 | 0.003 | 0.017 | 7.50E-10 |  | 0.015 | -0.009 | 0.536 |
| Chronotype | rs9364767 | T | G | 0.447 | 0.003 | 0.016 | 5.40E-09 |  | 0.015 | -0.019 | 0.214 |
| Chronotype | rs9521184 | T | C | 0.487 | 0.003 | 0.015 | 3.00E-08 |  | 0.015 | -0.006 | 0.679 |
| Chronotype | rs9573971 | A | G | 0.966 | 0.007 | 0.073 | 3.60E-22 |  | 0.041 | -0.049 | 0.231 |
| Chronotype | rs957501 | T | A | 0.336 | 0.003 | 0.015 | 3.00E-08 |  | 0.016 | -0.009 | 0.569 |
| Chronotype | rs9597250 | C | A | 0.810 | 0.003 | 0.021 | 6.90E-10 |  | 0.020 | 0.005 | 0.818 |
| Chronotype | rs975025 | C | T | 0.923 | 0.005 | 0.032 | 1.30E-10 |  | 0.025 | 0.033 | 0.187 |
| Chronotype | rs9817740 | A | G | 0.784 | 0.003 | 0.018 | 2.00E-08 |  | 0.017 | 0.000 | 0.996 |
| Chronotype | rs9831488 | A | G | 0.650 | 0.003 | -0.018 | 8.40E-11 |  | 0.016 | 0.019 | 0.229 |
| Chronotype | rs9964420 | C | A | 0.695 | 0.003 | 0.028 | 8.60E-22 |  | 0.016 | -0.048 | 0.003 |
| Daytime napping | rs10149986 | T | G | 0.815 | 0.002 | -0.011 | 4.40E-12 |  | 0.021 | -0.043 | 0.041 |
| Daytime napping | rs10152428 | G | C | 0.270 | 0.001 | -0.008 | 1.10E-08 |  | 0.016 | 0.000 | 0.988 |
| Daytime napping | rs10811438 | G | C | 0.607 | 0.001 | 0.007 | 3.20E-09 |  | 0.015 | 0.012 | 0.426 |
| Daytime napping | rs10835420 | T | A | 0.751 | 0.001 | 0.009 | 1.70E-10 |  | 0.021 | 0.053 | 0.010 |
| Daytime napping | rs10840017 | A | G | 0.767 | 0.001 | 0.009 | 2.00E-09 |  | 0.020 | -0.026 | 0.199 |
| Daytime napping | rs10875606 | C | A | 0.316 | 0.001 | 0.007 | 1.30E-08 |  | 0.017 | 0.008 | 0.630 |
| Daytime napping | rs10875622 | G | A | 0.425 | 0.001 | -0.010 | 1.30E-17 |  | 0.015 | 0.005 | 0.758 |
| Daytime napping | rs11071755 | G | A | 0.575 | 0.001 | 0.007 | 5.20E-09 |  | 0.016 | -0.015 | 0.338 |
| Daytime napping | rs112520848 | G | C | 0.614 | 0.001 | -0.007 | 1.80E-08 |  | 0.016 | 0.004 | 0.818 |
| Daytime napping | rs11258652 | C | A | 0.763 | 0.001 | 0.010 | 3.70E-13 |  | 0.016 | 0.006 | 0.732 |
| Daytime napping | rs11615756 | C | T | 0.595 | 0.001 | -0.018 | 1.40E-49 |  | 0.015 | 0.031 | 0.037 |
| Daytime napping | rs11682175 | T | C | 0.527 | 0.001 | -0.007 | 1.10E-08 |  | 0.015 | 0.009 | 0.533 |
| Daytime napping | rs11860072 | C | T | 0.553 | 0.001 | 0.009 | 2.80E-13 |  | 0.015 | 0.012 | 0.428 |
| Daytime napping | rs12042846 | T | C | 0.821 | 0.002 | -0.009 | 1.90E-08 |  | 0.020 | -0.022 | 0.260 |
| Daytime napping | rs12346996 | T | C | 0.272 | 0.001 | 0.008 | 5.00E-09 |  | 0.018 | 0.010 | 0.569 |
| Daytime napping | rs12451365 | T | C | 0.795 | 0.002 | -0.011 | 1.50E-12 |  | 0.018 | 0.023 | 0.190 |
| Daytime napping | rs12657723 | C | T | 0.678 | 0.001 | -0.008 | 2.00E-10 |  | 0.015 | -0.017 | 0.256 |
| Daytime napping | rs12992648 | A | G | 0.717 | 0.001 | 0.008 | 2.40E-08 |  | 0.017 | 0.009 | 0.607 |
| Daytime napping | rs13033444 | A | G | 0.717 | 0.001 | -0.010 | 3.60E-13 |  | 0.017 | -0.012 | 0.474 |
| Daytime napping | rs13150944 | A | G | 0.311 | 0.001 | -0.009 | 6.80E-11 |  | 0.017 | -0.004 | 0.794 |
| Daytime napping | rs13263535 | G | T | 0.531 | 0.001 | 0.007 | 1.60E-08 |  | 0.015 | -0.005 | 0.751 |
| Daytime napping | rs13284688 | T | C | 0.793 | 0.001 | -0.015 | 1.70E-23 |  | 0.020 | 0.018 | 0.373 |
| Daytime napping | rs140506252 | A | T | 0.978 | 0.004 | 0.023 | 4.30E-08 |  | 0.046 | -0.094 | 0.043 |
| Daytime napping | rs1546977 | A | G | 0.560 | 0.001 | -0.008 | 1.00E-11 |  | 0.015 | 0.005 | 0.738 |
| Daytime napping | rs1601440 | C | T | 0.279 | 0.001 | 0.009 | 1.90E-11 |  | 0.017 | -0.026 | 0.131 |
| Daytime napping | rs17158413 | G | A | 0.763 | 0.001 | -0.009 | 4.40E-11 |  | 0.019 | -0.045 | 0.019 |
| Daytime napping | rs17265513 | T | C | 0.801 | 0.002 | -0.009 | 2.00E-09 |  | 0.016 | -0.039 | 0.017 |
| Daytime napping | rs17502738 | T | C | 0.804 | 0.002 | 0.009 | 2.00E-08 |  | 0.019 | 0.019 | 0.322 |
| Daytime napping | rs1883048 | T | C | 0.476 | 0.001 | -0.008 | 1.80E-10 |  | 0.015 | 0.027 | 0.067 |
| Daytime napping | rs2033103 | C | T | 0.548 | 0.001 | -0.007 | 2.60E-09 |  | 0.015 | 0.019 | 0.199 |
| Daytime napping | rs2099810 | A | G | 0.503 | 0.001 | 0.008 | 2.80E-10 |  | 0.015 | 0.002 | 0.874 |
| Daytime napping | rs2202323 | G | C | 0.366 | 0.001 | 0.008 | 1.20E-09 |  | 0.016 | 0.011 | 0.475 |
| Daytime napping | rs2284015 | C | G | 0.741 | 0.001 | -0.008 | 4.10E-08 |  | 0.015 | 0.012 | 0.432 |
| Daytime napping | rs2370926 | T | C | 0.634 | 0.001 | 0.008 | 1.70E-10 |  | 0.018 | 0.026 | 0.145 |
| Daytime napping | rs2431108 | T | C | 0.671 | 0.001 | -0.013 | 7.70E-24 |  | 0.016 | -0.031 | 0.056 |
| Daytime napping | rs253666 | G | A | 0.235 | 0.001 | -0.008 | 1.80E-08 |  | 0.017 | 0.024 | 0.157 |
| Daytime napping | rs2653349 | A | G | 0.215 | 0.001 | 0.017 | 3.40E-29 |  | 0.019 | 0.038 | 0.042 |
| Daytime napping | rs2699869 | A | C | 0.454 | 0.001 | 0.007 | 3.10E-08 |  | 0.015 | 0.007 | 0.619 |
| Daytime napping | rs2769916 | G | A | 0.311 | 0.001 | -0.009 | 1.70E-11 |  | 0.015 | -0.008 | 0.578 |
| Daytime napping | rs2943023 | C | T | 0.580 | 0.001 | 0.007 | 7.40E-09 |  | 0.015 | -0.005 | 0.717 |
| Daytime napping | rs295278 | G | A | 0.605 | 0.001 | 0.008 | 2.20E-10 |  | 0.015 | 0.008 | 0.597 |
| Daytime napping | rs34262487 | C | A | 0.928 | 0.002 | 0.015 | 1.20E-09 |  | 0.037 | 0.024 | 0.517 |
| Daytime napping | rs35011311 | G | T | 0.734 | 0.001 | 0.009 | 4.40E-11 |  | 0.017 | -0.012 | 0.476 |
| Daytime napping | rs350785 | T | C | 0.115 | 0.002 | 0.013 | 1.50E-11 |  | 0.024 | -0.003 | 0.896 |
| Daytime napping | rs351776 | A | C | 0.450 | 0.001 | -0.008 | 8.40E-10 |  | 0.015 | -0.005 | 0.754 |
| Daytime napping | rs35851551 | A | G | 0.898 | 0.002 | 0.011 | 3.50E-08 |  | 0.032 | 0.049 | 0.117 |
| Daytime napping | rs3810484 | A | G | 0.556 | 0.001 | 0.007 | 2.20E-08 |  | 0.015 | -0.004 | 0.794 |
| Daytime napping | rs385199 | A | C | 0.772 | 0.001 | 0.021 | 7.50E-47 |  | 0.027 | -0.031 | 0.245 |
| Daytime napping | rs3935190 | G | A | 0.464 | 0.001 | -0.008 | 5.40E-11 |  | 0.015 | -0.017 | 0.263 |
| Daytime napping | rs4604518 | G | A | 0.546 | 0.001 | 0.007 | 1.40E-08 |  | 0.015 | -0.001 | 0.960 |
| Daytime napping | rs4653052 | C | A | 0.340 | 0.001 | -0.007 | 3.40E-08 |  | 0.016 | -0.013 | 0.417 |
| Daytime napping | rs467897 | G | A | 0.322 | 0.001 | 0.010 | 2.60E-13 |  | 0.016 | 0.055 | 0.000 |
| Daytime napping | rs4692709 | C | T | 0.455 | 0.001 | 0.007 | 2.40E-09 |  | 0.015 | -0.004 | 0.800 |
| Daytime napping | rs60222088 | C | A | 0.853 | 0.002 | 0.011 | 6.40E-11 |  | 0.018 | -0.027 | 0.124 |
| Daytime napping | rs60920123 | G | A | 0.567 | 0.001 | 0.008 | 4.50E-10 |  | 0.015 | 0.021 | 0.175 |
| Daytime napping | rs614987 | A | C | 0.386 | 0.001 | -0.011 | 7.50E-19 |  | 0.016 | -0.009 | 0.592 |
| Daytime napping | rs6919087 | T | G | 0.688 | 0.001 | 0.011 | 1.00E-16 |  | 0.016 | -0.011 | 0.505 |
| Daytime napping | rs73817091 | C | T | 0.958 | 0.003 | -0.017 | 3.80E-08 |  | 0.049 | 0.020 | 0.680 |
| Daytime napping | rs7422655 | C | T | 0.263 | 0.001 | 0.008 | 1.10E-08 |  | 0.016 | -0.034 | 0.040 |
| Daytime napping | rs7423968 | A | G | 0.571 | 0.001 | 0.008 | 4.80E-10 |  | 0.015 | 0.023 | 0.122 |
| Daytime napping | rs75022160 | C | T | 0.863 | 0.002 | 0.010 | 1.70E-08 |  | 0.025 | -0.013 | 0.615 |
| Daytime napping | rs76257331 | G | A | 0.802 | 0.002 | -0.009 | 1.20E-08 |  | 0.021 | 0.008 | 0.710 |
| Daytime napping | rs7697461 | A | T | 0.258 | 0.001 | -0.008 | 6.00E-09 |  | 0.016 | -0.027 | 0.096 |
| Daytime napping | rs77154532 | A | G | 0.642 | 0.001 | 0.008 | 1.40E-09 |  | 0.015 | 0.012 | 0.425 |
| Daytime napping | rs7814873 | C | T | 0.383 | 0.001 | 0.007 | 1.50E-08 |  | 0.016 | -0.029 | 0.067 |
| Daytime napping | rs785145 | T | G | 0.569 | 0.001 | -0.007 | 1.00E-08 |  | 0.015 | 0.014 | 0.346 |
| Daytime napping | rs7932966 | A | T | 0.237 | 0.001 | 0.009 | 1.10E-09 |  | 0.018 | 0.014 | 0.434 |
| Daytime napping | rs80163246 | T | C | 0.883 | 0.002 | -0.012 | 1.40E-10 |  | 0.022 | 0.011 | 0.598 |
| Daytime napping | rs908442 | A | T | 0.591 | 0.001 | 0.010 | 1.40E-16 |  | 0.016 | -0.004 | 0.822 |
| Daytime napping | rs910187 | G | A | 0.626 | 0.001 | 0.007 | 4.90E-09 |  | 0.015 | 0.022 | 0.160 |
| Daytime napping | rs9287862 | C | T | 0.087 | 0.002 | 0.012 | 2.00E-08 |  | 0.024 | 0.052 | 0.033 |
| Daytime napping | rs9309116 | C | T | 0.347 | 0.001 | -0.007 | 3.90E-09 |  | 0.015 | -0.017 | 0.261 |
| Daytime napping | rs9389556 | C | G | 0.739 | 0.001 | -0.008 | 9.70E-10 |  | 0.018 | -0.006 | 0.746 |
| Daytime napping | rs9460110 | T | C | 0.630 | 0.001 | -0.007 | 8.40E-09 |  | 0.016 | 0.011 | 0.492 |
| Daytime napping | rs9475168 | T | G | 0.781 | 0.001 | 0.009 | 1.90E-09 |  | 0.017 | -0.038 | 0.028 |
| Daytime napping | rs962247 | G | A | 0.523 | 0.001 | 0.008 | 5.70E-11 |  | 0.015 | -0.018 | 0.234 |
| Daytime napping | rs971415 | A | G | 0.878 | 0.002 | 0.011 | 1.20E-09 |  | 0.019 | -0.002 | 0.929 |
| Daytime napping | rs9939355 | C | T | 0.442 | 0.001 | 0.007 | 4.50E-09 |  | 0.015 | -0.017 | 0.283 |
| Daytime Sleepiness | rs11942333 | G | A | 0.676 | 0.001 | -0.006 | 3.80E-08 |  | 0.017 | 0.013 | 0.452 |
| Daytime Sleepiness | rs12153518 | A | G | 0.472 | 0.001 | 0.007 | 6.80E-11 |  | 0.015 | -0.009 | 0.546 |
| Daytime Sleepiness | rs13010456 | A | G | 0.595 | 0.001 | 0.008 | 2.10E-13 |  | 0.016 | -0.003 | 0.825 |
| Daytime Sleepiness | rs13097760 | A | C | 0.639 | 0.001 | -0.006 | 3.20E-08 |  | 0.016 | 0.002 | 0.882 |
| Daytime Sleepiness | rs147114641 | C | A | 0.774 | 0.001 | 0.008 | 1.70E-10 |  | 0.056 | -0.011 | 0.847 |
| Daytime Sleepiness | rs1566362 | T | C | 0.632 | 0.001 | 0.006 | 3.80E-09 |  | 0.015 | 0.007 | 0.633 |
| Daytime Sleepiness | rs17131124 | C | G | 0.912 | 0.002 | -0.011 | 1.70E-09 |  | 0.023 | -0.020 | 0.389 |
| Daytime Sleepiness | rs17356118 | A | G | 0.769 | 0.001 | -0.008 | 2.60E-10 |  | 0.020 | -0.042 | 0.036 |
| Daytime Sleepiness | rs1846644 | T | C | 0.591 | 0.001 | -0.011 | 2.50E-27 |  | 0.015 | 0.028 | 0.061 |
| Daytime Sleepiness | rs285793 | G | A | 0.461 | 0.001 | 0.007 | 7.90E-11 |  | 0.015 | -0.026 | 0.085 |
| Daytime Sleepiness | rs3122170 | C | A | 0.231 | 0.001 | 0.010 | 5.60E-15 |  | 0.020 | 0.034 | 0.085 |
| Daytime Sleepiness | rs4765939 | G | C | 0.583 | 0.001 | -0.006 | 2.00E-09 |  | 0.016 | -0.009 | 0.571 |
| Daytime Sleepiness | rs501701 | A | G | 0.531 | 0.001 | 0.006 | 6.50E-09 |  | 0.015 | 0.013 | 0.394 |
| Daytime Sleepiness | rs55818482 | T | C | 0.785 | 0.001 | -0.010 | 1.40E-14 |  | 0.020 | 0.010 | 0.606 |
| Daytime Sleepiness | rs55960940 | T | C | 0.822 | 0.001 | 0.008 | 2.00E-08 |  | 0.018 | 0.026 | 0.153 |
| Daytime Sleepiness | rs62519825 | T | C | 0.887 | 0.002 | -0.009 | 3.80E-09 |  | 0.021 | -0.023 | 0.286 |
| Daytime Sleepiness | rs6897863 | A | C | 0.584 | 0.001 | 0.006 | 7.60E-10 |  | 0.015 | -0.008 | 0.580 |
| Daytime Sleepiness | rs6923811 | T | C | 0.679 | 0.001 | 0.007 | 9.10E-10 |  | 0.019 | -0.011 | 0.576 |
| Daytime Sleepiness | rs7476897 | G | A | 0.679 | 0.001 | 0.007 | 2.70E-11 |  | 0.018 | 0.008 | 0.639 |
| Daytime Sleepiness | rs7598712 | G | T | 0.555 | 0.001 | 0.006 | 2.20E-08 |  | 0.015 | -0.005 | 0.727 |
| Daytime Sleepiness | rs7837226 | A | G | 0.473 | 0.001 | -0.006 | 2.00E-08 |  | 0.015 | -0.025 | 0.087 |
| Daytime Sleepiness | rs8015449 | A | G | 0.539 | 0.001 | 0.006 | 1.90E-09 |  | 0.015 | 0.014 | 0.336 |
| Daytime Sleepiness | rs825127 | T | G | 0.531 | 0.001 | 0.006 | 9.50E-09 |  | 0.015 | 0.002 | 0.885 |
| Daytime Sleepiness | rs843372 | C | T | 0.230 | 0.001 | 0.008 | 2.20E-11 |  | 0.016 | 0.013 | 0.415 |
| Daytime Sleepiness | rs886114 | C | T | 0.357 | 0.001 | 0.006 | 1.90E-08 |  | 0.016 | -0.011 | 0.517 |
| Insomnia | rs10156602 | A | G | 0.638 | 0.001 | 0.010 | 3.40E-12 |  | 0.015 | -0.019 | 0.212 |
| Insomnia | rs10947690 | A | G | 0.738 | 0.002 | -0.009 | 3.50E-08 |  | 0.018 | -0.005 | 0.772 |
| Insomnia | rs11097861 | A | G | 0.284 | 0.002 | -0.009 | 1.70E-09 |  | 0.016 | -0.024 | 0.134 |
| Insomnia | rs11184946 | C | T | 0.583 | 0.001 | -0.009 | 2.90E-10 |  | 0.015 | 0.007 | 0.643 |
| Insomnia | rs113851554 | G | T | 0.943 | 0.003 | -0.041 | 1.30E-41 |  | 0.029 | -0.049 | 0.090 |
| Insomnia | rs11673344 | A | G | 0.620 | 0.001 | -0.009 | 9.00E-10 |  | 0.016 | 0.000 | 0.982 |
| Insomnia | rs11804386 | G | A | 0.667 | 0.001 | -0.008 | 2.60E-08 |  | 0.016 | 0.008 | 0.617 |
| Insomnia | rs12405761 | A | C | 0.571 | 0.001 | 0.009 | 2.60E-11 |  | 0.016 | 0.024 | 0.133 |
| Insomnia | rs1327938 | T | C | 0.346 | 0.001 | -0.009 | 3.80E-10 |  | 0.015 | -0.012 | 0.432 |
| Insomnia | rs1592757 | G | C | 0.644 | 0.001 | -0.009 | 4.60E-10 |  | 0.016 | -0.015 | 0.325 |
| Insomnia | rs17139246 | T | C | 0.611 | 0.001 | -0.008 | 4.10E-08 |  | 0.015 | -0.005 | 0.714 |
| Insomnia | rs17151854 | G | T | 0.847 | 0.002 | -0.011 | 2.40E-08 |  | 0.022 | 0.010 | 0.651 |
| Insomnia | rs17669584 | A | G | 0.805 | 0.002 | -0.010 | 3.60E-08 |  | 0.020 | -0.006 | 0.772 |
| Insomnia | rs1841625 | A | G | 0.568 | 0.001 | -0.008 | 3.60E-08 |  | 0.015 | 0.003 | 0.855 |
| Insomnia | rs1942262 | G | A | 0.708 | 0.002 | -0.011 | 1.10E-13 |  | 0.017 | -0.011 | 0.517 |
| Insomnia | rs2062113 | T | C | 0.429 | 0.001 | 0.009 | 1.90E-10 |  | 0.015 | 0.004 | 0.806 |
| Insomnia | rs2296580 | G | T | 0.702 | 0.002 | 0.010 | 8.70E-12 |  | 0.017 | 0.012 | 0.456 |
| Insomnia | rs2956278 | A | G | 0.785 | 0.002 | -0.010 | 1.30E-08 |  | 0.020 | 0.008 | 0.702 |
| Insomnia | rs324017 | A | C | 0.295 | 0.002 | 0.010 | 1.10E-10 |  | 0.016 | -0.043 | 0.006 |
| Insomnia | rs3824081 | T | C | 0.476 | 0.001 | 0.008 | 1.10E-08 |  | 0.015 | -0.018 | 0.217 |
| Insomnia | rs4577309 | A | G | 0.469 | 0.001 | 0.008 | 3.70E-09 |  | 0.015 | 0.001 | 0.922 |
| Insomnia | rs4886860 | G | C | 0.234 | 0.002 | 0.011 | 6.10E-12 |  | 0.016 | -0.001 | 0.945 |
| Insomnia | rs62158170 | A | G | 0.785 | 0.002 | 0.012 | 5.70E-13 |  | 0.018 | -0.003 | 0.858 |
| Insomnia | rs6593005 | A | G | 0.259 | 0.002 | -0.009 | 8.60E-09 |  | 0.016 | -0.007 | 0.650 |
| Insomnia | rs6664467 | G | A | 0.864 | 0.002 | 0.011 | 4.50E-08 |  | 0.021 | -0.007 | 0.722 |
| Insomnia | rs68094047 | C | T | 0.750 | 0.002 | -0.009 | 3.30E-09 |  | 0.020 | -0.012 | 0.531 |
| Insomnia | rs7711696 | G | T | 0.695 | 0.002 | -0.010 | 9.90E-12 |  | 0.016 | -0.013 | 0.409 |
| Insomnia | rs9845387 | C | A | 0.959 | 0.004 | 0.020 | 2.10E-08 |  | 0.033 | 0.062 | 0.060 |
| Long sleep duration | rs10899257 | G | A | 0.856 | 0.001 | -0.006 | 4.60E-08 |  | 0.021 | -0.004 | 0.842 |
| Long sleep duration | rs146467757 | G | T | 0.772 | 0.001 | 0.006 | 8.10E-11 |  | 0.048 | -0.102 | 0.035 |
| Long sleep duration | rs147114641 | C | A | 0.774 | 0.001 | 0.006 | 4.90E-11 |  | 0.056 | -0.011 | 0.847 |
| Long sleep duration | rs6737318 | A | G | 0.778 | 0.001 | -0.006 | 3.40E-13 |  | 0.018 | 0.001 | 0.963 |
| Long sleep duration | rs7534398 | T | A | 0.799 | 0.001 | -0.005 | 2.10E-08 |  | 0.019 | -0.010 | 0.580 |
| Long sleep duration | rs75458655 | C | T | 0.977 | 0.002 | -0.017 | 5.40E-12 |  | 0.056 | 0.034 | 0.539 |
| Morning person | rs10067113 | C | T | 0.381 | 0.005 | -0.028 | 2.50E-09 |  | 0.016 | -0.036 | 0.021 |
| Morning person | rs10123584 | G | A | 0.293 | 0.005 | 0.027 | 4.00E-08 |  | 0.018 | 0.028 | 0.112 |
| Morning person | rs10149448 | A | G | 0.604 | 0.005 | 0.027 | 5.60E-09 |  | 0.015 | -0.017 | 0.263 |
| Morning person | rs10196909 | C | A | 0.482 | 0.005 | 0.032 | 6.20E-13 |  | 0.015 | -0.009 | 0.523 |
| Morning person | rs1027742 | A | G | 0.738 | 0.005 | 0.031 | 3.60E-09 |  | 0.016 | 0.007 | 0.678 |
| Morning person | rs10495976 | A | T | 0.610 | 0.005 | -0.028 | 8.00E-10 |  | 0.015 | 0.003 | 0.853 |
| Morning person | rs10818834 | T | C | 0.733 | 0.005 | 0.032 | 4.20E-10 |  | 0.016 | -0.008 | 0.592 |
| Morning person | rs10976942 | C | A | 0.917 | 0.008 | -0.049 | 1.00E-09 |  | 0.033 | 0.025 | 0.454 |
| Morning person | rs11032362 | G | A | 0.909 | 0.008 | -0.067 | 7.30E-18 |  | 0.032 | 0.009 | 0.777 |
| Morning person | rs11152350 | A | C | 0.471 | 0.005 | -0.028 | 6.40E-10 |  | 0.015 | -0.021 | 0.172 |
| Morning person | rs11174781 | T | C | 0.878 | 0.007 | 0.048 | 3.90E-12 |  | 0.020 | 0.018 | 0.372 |
| Morning person | rs1144566 | T | C | 0.030 | 0.013 | 0.171 | 2.80E-38 |  | 0.059 | -0.050 | 0.394 |
| Morning person | rs114870822 | G | A | 0.987 | 0.020 | -0.108 | 3.40E-08 |  | 0.067 | 0.063 | 0.343 |
| Morning person | rs115774037 | T | C | 0.974 | 0.014 | -0.079 | 3.50E-08 |  | 0.042 | -0.021 | 0.615 |
| Morning person | rs11580135 | T | C | 0.317 | 0.005 | -0.028 | 1.30E-08 |  | 0.016 | -0.014 | 0.374 |
| Morning person | rs11587758 | G | A | 0.604 | 0.005 | -0.035 | 3.80E-14 |  | 0.015 | 0.017 | 0.247 |
| Morning person | rs11588913 | G | A | 0.601 | 0.005 | 0.025 | 2.50E-08 |  | 0.015 | -0.012 | 0.441 |
| Morning person | rs11645898 | T | C | 0.833 | 0.006 | 0.036 | 1.80E-09 |  | 0.018 | -0.039 | 0.028 |
| Morning person | rs11670534 | C | T | 0.834 | 0.006 | 0.033 | 4.70E-08 |  | 0.022 | -0.043 | 0.049 |
| Morning person | rs11679484 | C | A | 0.626 | 0.005 | -0.030 | 1.60E-10 |  | 0.016 | -0.028 | 0.081 |
| Morning person | rs11786306 | G | C | 0.646 | 0.005 | -0.029 | 7.00E-10 |  | 0.015 | 0.029 | 0.057 |
| Morning person | rs11841335 | G | A | 0.746 | 0.005 | 0.028 | 4.00E-08 |  | 0.017 | -0.024 | 0.171 |
| Morning person | rs12927162 | A | G | 0.722 | 0.005 | 0.046 | 2.00E-20 |  | 0.016 | 0.006 | 0.710 |
| Morning person | rs12969848 | C | T | 0.470 | 0.005 | -0.036 | 2.60E-15 |  | 0.015 | -0.003 | 0.836 |
| Morning person | rs13065394 | G | T | 0.712 | 0.005 | 0.030 | 2.20E-09 |  | 0.016 | 0.010 | 0.530 |
| Morning person | rs13255030 | A | G | 0.585 | 0.005 | 0.026 | 1.50E-08 |  | 0.015 | -0.010 | 0.531 |
| Morning person | rs13269289 | G | A | 0.673 | 0.005 | -0.028 | 5.50E-09 |  | 0.016 | 0.002 | 0.904 |
| Morning person | rs138964083 | C | T | 0.941 | 0.010 | -0.059 | 1.00E-09 |  | 0.037 | 0.041 | 0.270 |
| Morning person | rs1470764 | G | A | 0.387 | 0.005 | 0.029 | 5.90E-10 |  | 0.015 | -0.024 | 0.110 |
| Morning person | rs1494185 | G | A | 0.726 | 0.005 | 0.029 | 1.10E-08 |  | 0.016 | -0.005 | 0.772 |
| Morning person | rs149611468 | T | C | 0.988 | 0.021 | 0.120 | 1.30E-08 |  | 0.050 | -0.062 | 0.214 |
| Morning person | rs1524472 | A | G | 0.441 | 0.005 | 0.025 | 2.10E-08 |  | 0.015 | 0.017 | 0.246 |
| Morning person | rs17374439 | C | T | 0.804 | 0.006 | -0.056 | 1.20E-22 |  | 0.017 | 0.008 | 0.654 |
| Morning person | rs17575798 | G | A | 0.807 | 0.006 | 0.039 | 7.80E-12 |  | 0.018 | -0.036 | 0.053 |
| Morning person | rs17682747 | G | A | 0.767 | 0.005 | -0.031 | 8.70E-09 |  | 0.017 | 0.001 | 0.940 |
| Morning person | rs1947198 | C | T | 0.878 | 0.007 | -0.040 | 3.20E-09 |  | 0.020 | 0.003 | 0.891 |
| Morning person | rs202157 | C | T | 0.299 | 0.005 | 0.040 | 1.30E-15 |  | 0.018 | -0.019 | 0.304 |
| Morning person | rs2072727 | T | C | 0.436 | 0.005 | 0.028 | 5.60E-10 |  | 0.015 | -0.021 | 0.166 |
| Morning person | rs2102506 | G | A | 0.361 | 0.005 | 0.029 | 8.30E-10 |  | 0.015 | -0.005 | 0.735 |
| Morning person | rs2239626 | T | C | 0.695 | 0.005 | -0.034 | 5.00E-12 |  | 0.018 | 0.007 | 0.697 |
| Morning person | rs231398 | G | A | 0.838 | 0.006 | 0.035 | 5.80E-09 |  | 0.019 | 0.013 | 0.483 |
| Morning person | rs2467109 | T | A | 0.718 | 0.005 | -0.028 | 3.90E-08 |  | 0.018 | 0.002 | 0.890 |
| Morning person | rs2518022 | T | C | 0.084 | 0.008 | 0.063 | 7.60E-15 |  | 0.031 | -0.064 | 0.037 |
| Morning person | rs2653343 | T | A | 0.215 | 0.005 | 0.061 | 5.20E-29 |  | 0.019 | 0.037 | 0.044 |
| Morning person | rs28380327 | A | T | 0.629 | 0.005 | 0.029 | 7.80E-10 |  | 0.016 | 0.007 | 0.658 |
| Morning person | rs2842638 | T | G | 0.577 | 0.005 | 0.031 | 1.90E-11 |  | 0.015 | 0.003 | 0.825 |
| Morning person | rs28458909 | C | T | 0.876 | 0.007 | 0.068 | 4.70E-23 |  | 0.019 | -0.022 | 0.244 |
| Morning person | rs28634184 | C | T | 0.749 | 0.005 | 0.030 | 1.30E-08 |  | 0.018 | 0.019 | 0.285 |
| Morning person | rs2893787 | G | A | 0.255 | 0.005 | 0.029 | 3.50E-08 |  | 0.017 | -0.002 | 0.913 |
| Morning person | rs2910032 | C | T | 0.482 | 0.005 | -0.034 | 1.10E-13 |  | 0.015 | 0.010 | 0.516 |
| Morning person | rs2949923 | A | G | 0.539 | 0.005 | -0.027 | 4.20E-09 |  | 0.015 | 0.013 | 0.386 |
| Morning person | rs308521 | T | C | 0.603 | 0.005 | 0.028 | 1.50E-09 |  | 0.016 | 0.014 | 0.378 |
| Morning person | rs34581681 | G | A | 0.839 | 0.006 | 0.034 | 4.20E-08 |  | 0.021 | 0.014 | 0.514 |
| Morning person | rs34619169 | G | A | 0.692 | 0.005 | -0.027 | 1.50E-08 |  | 0.017 | 0.027 | 0.115 |
| Morning person | rs34627176 | G | A | 0.786 | 0.006 | -0.030 | 3.60E-08 |  | 0.019 | -0.014 | 0.442 |
| Morning person | rs34875688 | T | A | 0.767 | 0.005 | 0.032 | 1.10E-09 |  | 0.018 | -0.006 | 0.738 |
| Morning person | rs35653190 | C | T | 0.772 | 0.005 | 0.030 | 2.00E-08 |  | 0.019 | -0.012 | 0.543 |
| Morning person | rs3760185 | C | T | 0.752 | 0.005 | 0.037 | 2.10E-12 |  | 0.019 | 0.016 | 0.403 |
| Morning person | rs3767240 | T | C | 0.618 | 0.005 | -0.031 | 7.30E-12 |  | 0.015 | 0.001 | 0.928 |
| Morning person | rs3850174 | T | A | 0.743 | 0.005 | 0.032 | 1.40E-09 |  | 0.018 | 0.010 | 0.576 |
| Morning person | rs3877930 | A | G | 0.986 | 0.019 | -0.112 | 3.00E-09 |  | 0.048 | 0.067 | 0.157 |
| Morning person | rs4239386 | T | A | 0.665 | 0.005 | 0.034 | 4.20E-13 |  | 0.015 | -0.025 | 0.101 |
| Morning person | rs4339281 | A | G | 0.873 | 0.007 | 0.039 | 5.30E-09 |  | 0.022 | -0.006 | 0.777 |
| Morning person | rs4752593 | G | C | 0.372 | 0.005 | 0.027 | 9.80E-09 |  | 0.015 | -0.003 | 0.828 |
| Morning person | rs4822107 | G | A | 0.494 | 0.005 | -0.028 | 8.70E-10 |  | 0.015 | 0.004 | 0.779 |
| Morning person | rs4936291 | A | G | 0.611 | 0.005 | -0.028 | 9.40E-09 |  | 0.015 | 0.026 | 0.085 |
| Morning person | rs520954 | A | G | 0.673 | 0.005 | -0.042 | 1.60E-18 |  | 0.017 | 0.010 | 0.551 |
| Morning person | rs60194061 | G | A | 0.730 | 0.005 | -0.033 | 8.60E-11 |  | 0.020 | -0.050 | 0.011 |
| Morning person | rs60616179 | A | G | 0.945 | 0.010 | 0.056 | 9.20E-09 |  | 0.025 | 0.008 | 0.762 |
| Morning person | rs62046253 | C | T | 0.656 | 0.005 | -0.030 | 1.40E-10 |  | 0.015 | -0.006 | 0.681 |
| Morning person | rs62082401 | C | G | 0.809 | 0.006 | -0.043 | 1.20E-14 |  | 0.018 | 0.014 | 0.441 |
| Morning person | rs62553781 | C | T | 0.965 | 0.012 | 0.083 | 3.00E-11 |  | 0.068 | 0.073 | 0.285 |
| Morning person | rs6537834 | T | C | 0.385 | 0.005 | -0.025 | 3.50E-08 |  | 0.015 | 0.029 | 0.054 |
| Morning person | rs6599694 | G | T | 0.656 | 0.005 | 0.027 | 2.10E-08 |  | 0.016 | 0.002 | 0.919 |
| Morning person | rs6656331 | C | T | 0.476 | 0.005 | 0.027 | 1.50E-09 |  | 0.015 | 0.019 | 0.192 |
| Morning person | rs6744983 | G | T | 0.623 | 0.005 | -0.025 | 4.90E-08 |  | 0.016 | -0.029 | 0.068 |
| Morning person | rs6967481 | C | T | 0.503 | 0.005 | -0.030 | 1.90E-11 |  | 0.015 | -0.009 | 0.559 |
| Morning person | rs7001604 | T | C | 0.613 | 0.005 | -0.032 | 7.30E-12 |  | 0.016 | 0.000 | 0.981 |
| Morning person | rs72632979 | A | G | 0.829 | 0.006 | 0.035 | 4.00E-09 |  | 0.029 | -0.028 | 0.341 |
| Morning person | rs72829936 | G | A | 0.836 | 0.006 | -0.035 | 5.50E-09 |  | 0.021 | 0.012 | 0.558 |
| Morning person | rs7302062 | T | C | 0.553 | 0.005 | 0.032 | 5.20E-12 |  | 0.015 | -0.006 | 0.699 |
| Morning person | rs7304278 | A | G | 0.275 | 0.005 | -0.033 | 9.60E-11 |  | 0.017 | -0.001 | 0.939 |
| Morning person | rs73606718 | G | A | 0.879 | 0.007 | 0.040 | 5.70E-09 |  | 0.023 | 0.038 | 0.102 |
| Morning person | rs75120545 | C | T | 0.970 | 0.014 | -0.097 | 2.90E-12 |  | 0.036 | -0.007 | 0.848 |
| Morning person | rs7547493 | A | G | 0.822 | 0.006 | -0.058 | 2.50E-23 |  | 0.017 | -0.003 | 0.839 |
| Morning person | rs75650221 | C | T | 0.962 | 0.012 | -0.065 | 3.30E-08 |  | 0.042 | 0.024 | 0.561 |
| Morning person | rs7602425 | C | T | 0.924 | 0.009 | -0.054 | 3.20E-10 |  | 0.038 | -0.072 | 0.055 |
| Morning person | rs769066 | T | C | 0.816 | 0.006 | -0.033 | 1.90E-08 |  | 0.021 | -0.002 | 0.938 |
| Morning person | rs7691121 | C | G | 0.768 | 0.005 | 0.034 | 1.00E-10 |  | 0.017 | -0.010 | 0.569 |
| Morning person | rs77008212 | A | G | 0.913 | 0.008 | 0.083 | 1.10E-24 |  | 0.021 | 0.005 | 0.802 |
| Morning person | rs7701529 | A | T | 0.238 | 0.005 | -0.030 | 1.70E-08 |  | 0.019 | 0.028 | 0.147 |
| Morning person | rs7735794 | G | A | 0.776 | 0.006 | -0.031 | 4.50E-08 |  | 0.017 | -0.011 | 0.509 |
| Morning person | rs778147 | C | A | 0.367 | 0.005 | 0.030 | 4.60E-10 |  | 0.015 | 0.027 | 0.072 |
| Morning person | rs786406 | A | G | 0.298 | 0.005 | -0.033 | 1.00E-11 |  | 0.015 | 0.004 | 0.795 |
| Morning person | rs7959983 | T | C | 0.596 | 0.005 | -0.032 | 4.70E-12 |  | 0.016 | -0.011 | 0.473 |
| Morning person | rs9573971 | A | G | 0.966 | 0.012 | 0.108 | 4.90E-18 |  | 0.041 | -0.049 | 0.231 |
| Morning person | rs957501 | T | A | 0.336 | 0.005 | 0.026 | 3.00E-08 |  | 0.016 | -0.009 | 0.569 |
| Morning person | rs9597241 | A | C | 0.811 | 0.006 | 0.032 | 2.70E-08 |  | 0.020 | 0.004 | 0.823 |
| Morning person | rs9964420 | C | A | 0.696 | 0.005 | 0.041 | 2.40E-16 |  | 0.016 | -0.048 | 0.003 |
| Short sleep duration | rs1229762 | C | T | 0.335 | 0.001 | -0.007 | 1.00E-12 |  | 0.016 | -0.031 | 0.053 |
| Short sleep duration | rs12518468 | T | C | 0.672 | 0.001 | -0.006 | 8.50E-09 |  | 0.016 | 0.022 | 0.155 |
| Short sleep duration | rs12567114 | G | A | 0.725 | 0.001 | 0.006 | 4.10E-09 |  | 0.017 | 0.005 | 0.767 |
| Short sleep duration | rs12661667 | C | T | 0.737 | 0.001 | -0.006 | 2.80E-08 |  | 0.017 | 0.017 | 0.313 |
| Short sleep duration | rs12963463 | C | T | 0.299 | 0.001 | 0.007 | 1.90E-11 |  | 0.017 | -0.016 | 0.340 |
| Short sleep duration | rs1380703 | A | G | 0.616 | 0.001 | -0.007 | 1.60E-11 |  | 0.015 | -0.019 | 0.222 |
| Short sleep duration | rs17388803 | A | C | 0.894 | 0.002 | -0.010 | 6.50E-10 |  | 0.028 | -0.007 | 0.801 |
| Short sleep duration | rs205024 | C | T | 0.617 | 0.001 | 0.006 | 2.70E-08 |  | 0.015 | -0.012 | 0.412 |
| Short sleep duration | rs2863957 | C | A | 0.782 | 0.001 | 0.010 | 2.60E-18 |  | 0.018 | 0.001 | 0.946 |
| Short sleep duration | rs4585442 | A | G | 0.689 | 0.001 | -0.006 | 8.10E-10 |  | 0.016 | -0.012 | 0.445 |
| Short sleep duration | rs5757675 | G | T | 0.260 | 0.001 | 0.006 | 2.70E-09 |  | 0.017 | 0.024 | 0.149 |
| Short sleep duration | rs59779556 | T | G | 0.554 | 0.001 | 0.005 | 2.00E-08 |  | 0.015 | -0.014 | 0.349 |
| Short sleep duration | rs60882754 | A | T | 0.939 | 0.002 | 0.011 | 1.80E-08 |  | 0.048 | -0.007 | 0.887 |
| Short sleep duration | rs7524118 | T | C | 0.292 | 0.001 | -0.006 | 4.90E-08 |  | 0.016 | -0.006 | 0.699 |
| Short sleep duration | rs7939345 | T | G | 0.208 | 0.001 | 0.006 | 4.00E-08 |  | 0.021 | -0.013 | 0.538 |
| Short sleep duration | rs9321171 | C | T | 0.540 | 0.001 | 0.005 | 4.20E-08 |  | 0.015 | 0.024 | 0.116 |
| Obstructive sleep apnea | rs10507084 | T | C | 0.179 | 0.010 | 0.065 | 8.23E-11 |  | 0.019 | 0.002 | 0.906 |
| Obstructive sleep apnea | rs113955098 | A | G | 0.067 | 0.016 | -0.099 | 3.38E-10 |  | 0.030 | -0.057 | 0.059 |
| Obstructive sleep apnea | rs114106239 | T | C | 0.037 | 0.021 | -0.121 | 1.19E-08 |  | 0.040 | 0.014 | 0.720 |
| Obstructive sleep apnea | rs1228509 | C | A | 0.656 | 0.008 | 0.047 | 6.41E-09 |  | 0.016 | 0.008 | 0.613 |
| Obstructive sleep apnea | rs13114985 | G | T | 0.338 | 0.008 | 0.046 | 2.01E-08 |  | 0.016 | -0.008 | 0.604 |
| Obstructive sleep apnea | rs140896965 | T | C | 0.049 | 0.018 | -0.113 | 1.11E-09 |  | 0.035 | -0.027 | 0.431 |
| Obstructive sleep apnea | rs2016950 | T | C | 0.158 | 0.011 | -0.059 | 4.13E-08 |  | 0.020 | -0.024 | 0.241 |
| Obstructive sleep apnea | rs59333125 | C | A | 0.081 | 0.014 | -0.082 | 1.32E-08 |  | 0.027 | 0.022 | 0.414 |
| Obstructive sleep apnea | rs60700772 | C | T | 0.221 | 0.009 | 0.052 | 2.20E-08 |  | 0.018 | 0.004 | 0.805 |
| Obstructive sleep apnea | rs6484367 | A | G | 0.503 | 0.008 | 0.047 | 1.13E-09 |  | 0.015 | 0.051 | 0.001 |
| Obstructive sleep apnea | rs679880 | A | G | 0.745 | 0.009 | 0.050 | 2.53E-08 |  | 0.017 | 0.007 | 0.675 |
| Obstructive sleep apnea | rs76229479 | C | A | 0.099 | 0.013 | -0.078 | 2.64E-09 |  | 0.025 | 0.006 | 0.805 |
| Sleep duration | rs10173260 | T | C | 0.394 | 0.002 | -0.013 | 2.90E-08 |  | 0.015 | 0.017 | 0.258 |
| Sleep duration | rs1079727 | T | C | 0.842 | 0.003 | -0.018 | 5.30E-09 |  | 0.019 | -0.023 | 0.219 |
| Sleep duration | rs10973207 | G | T | 0.842 | 0.003 | -0.020 | 6.00E-11 |  | 0.017 | -0.031 | 0.071 |
| Sleep duration | rs11039544 | G | A | 0.838 | 0.003 | 0.018 | 1.90E-09 |  | 0.022 | 0.000 | 0.985 |
| Sleep duration | rs112230981 | A | G | 0.950 | 0.005 | 0.032 | 2.20E-09 |  | 0.045 | 0.006 | 0.895 |
| Sleep duration | rs113113059 | T | C | 0.780 | 0.003 | 0.016 | 8.40E-09 |  | 0.019 | -0.054 | 0.005 |
| Sleep duration | rs11567976 | C | T | 0.429 | 0.002 | -0.013 | 2.10E-08 |  | 0.015 | 0.020 | 0.186 |
| Sleep duration | rs11621908 | C | T | 0.917 | 0.004 | 0.024 | 5.60E-09 |  | 0.031 | -0.018 | 0.558 |
| Sleep duration | rs11643715 | C | G | 0.709 | 0.002 | -0.014 | 3.20E-08 |  | 0.018 | 0.004 | 0.807 |
| Sleep duration | rs11885663 | C | T | 0.752 | 0.003 | -0.016 | 8.60E-10 |  | 0.019 | -0.025 | 0.185 |
| Sleep duration | rs12567114 | G | A | 0.724 | 0.003 | -0.015 | 4.30E-09 |  | 0.017 | 0.005 | 0.767 |
| Sleep duration | rs12607679 | T | C | 0.738 | 0.003 | 0.020 | 8.30E-15 |  | 0.018 | 0.014 | 0.411 |
| Sleep duration | rs12791153 | A | T | 0.919 | 0.004 | -0.024 | 1.90E-08 |  | 0.028 | 0.008 | 0.773 |
| Sleep duration | rs147114641 | C | A | 0.774 | 0.003 | 0.016 | 3.10E-09 |  | 0.056 | -0.011 | 0.847 |
| Sleep duration | rs151014368 | G | A | 0.794 | 0.003 | -0.016 | 9.10E-09 |  | 0.017 | -0.028 | 0.105 |
| Sleep duration | rs1517572 | A | C | 0.419 | 0.002 | -0.015 | 1.50E-10 |  | 0.016 | 0.006 | 0.715 |
| Sleep duration | rs1553132 | A | G | 0.742 | 0.003 | -0.015 | 2.50E-08 |  | 0.017 | 0.015 | 0.388 |
| Sleep duration | rs1776776 | T | C | 0.874 | 0.003 | 0.020 | 4.90E-09 |  | 0.019 | -0.017 | 0.367 |
| Sleep duration | rs1939455 | G | T | 0.879 | 0.004 | 0.020 | 1.20E-08 |  | 0.027 | -0.032 | 0.246 |
| Sleep duration | rs205024 | C | T | 0.616 | 0.002 | -0.014 | 3.90E-09 |  | 0.015 | -0.012 | 0.412 |
| Sleep duration | rs2072727 | T | C | 0.436 | 0.002 | 0.013 | 7.90E-09 |  | 0.015 | -0.021 | 0.166 |
| Sleep duration | rs2079070 | C | G | 0.265 | 0.003 | 0.018 | 7.50E-12 |  | 0.019 | -0.022 | 0.241 |
| Sleep duration | rs2231265 | A | G | 0.228 | 0.003 | -0.015 | 2.70E-08 |  | 0.019 | -0.033 | 0.082 |
| Sleep duration | rs2696429 | G | A | 0.774 | 0.003 | 0.017 | 4.00E-10 |  | 0.027 | -0.026 | 0.342 |
| Sleep duration | rs2717076 | C | T | 0.373 | 0.002 | -0.018 | 3.10E-15 |  | 0.015 | 0.019 | 0.213 |
| Sleep duration | rs3027234 | C | T | 0.773 | 0.003 | 0.015 | 2.30E-08 |  | 0.020 | -0.030 | 0.132 |
| Sleep duration | rs3095508 | C | A | 0.594 | 0.002 | 0.015 | 3.10E-11 |  | 0.015 | 0.004 | 0.783 |
| Sleep duration | rs34354917 | C | A | 0.710 | 0.003 | 0.014 | 3.90E-08 |  | 0.017 | 0.006 | 0.745 |
| Sleep duration | rs35531607 | T | C | 0.526 | 0.002 | -0.013 | 1.50E-08 |  | 0.015 | 0.010 | 0.491 |
| Sleep duration | rs35662245 | T | A | 0.661 | 0.002 | -0.015 | 1.40E-09 |  | 0.016 | 0.015 | 0.319 |
| Sleep duration | rs365663 | A | G | 0.546 | 0.002 | 0.015 | 1.00E-10 |  | 0.015 | 0.029 | 0.054 |
| Sleep duration | rs374153 | C | T | 0.158 | 0.003 | 0.018 | 9.10E-09 |  | 0.018 | 0.024 | 0.179 |
| Sleep duration | rs465700 | C | G | 0.108 | 0.004 | 0.023 | 1.40E-09 |  | 0.022 | -0.047 | 0.033 |
| Sleep duration | rs4767550 | A | G | 0.586 | 0.002 | -0.014 | 6.30E-10 |  | 0.015 | 0.029 | 0.053 |
| Sleep duration | rs55658675 | C | T | 0.645 | 0.002 | 0.013 | 2.00E-08 |  | 0.015 | 0.000 | 0.997 |
| Sleep duration | rs61985058 | C | T | 0.857 | 0.003 | -0.019 | 1.30E-08 |  | 0.025 | -0.017 | 0.498 |
| Sleep duration | rs62120041 | T | C | 0.934 | 0.005 | 0.026 | 9.60E-09 |  | 0.027 | -0.054 | 0.047 |
| Sleep duration | rs6575005 | T | C | 0.758 | 0.003 | 0.016 | 4.40E-09 |  | 0.018 | -0.005 | 0.783 |
| Sleep duration | rs7198661 | T | C | 0.502 | 0.002 | 0.013 | 3.90E-09 |  | 0.015 | 0.008 | 0.616 |
| Sleep duration | rs7556815 | G | A | 0.781 | 0.003 | -0.041 | 1.30E-49 |  | 0.018 | 0.001 | 0.954 |
| Sleep duration | rs7644809 | T | C | 0.422 | 0.002 | 0.013 | 1.60E-08 |  | 0.015 | 0.006 | 0.678 |
| Sleep duration | rs7806045 | T | C | 0.755 | 0.003 | 0.015 | 1.40E-08 |  | 0.016 | -0.024 | 0.150 |
| Sleep duration | rs7915425 | T | C | 0.175 | 0.003 | 0.019 | 2.00E-10 |  | 0.020 | 0.028 | 0.159 |
| Sleep duration | rs8038326 | A | G | 0.727 | 0.003 | 0.016 | 2.80E-10 |  | 0.016 | -0.022 | 0.168 |
| Sleep duration | rs915416 | C | G | 0.290 | 0.002 | 0.019 | 9.90E-15 |  | 0.016 | -0.003 | 0.867 |
| Sleep duration | rs9345234 | A | C | 0.422 | 0.002 | -0.013 | 1.80E-08 |  | 0.015 | 0.008 | 0.589 |
| Sleep duration | rs9382445 | T | C | 0.623 | 0.002 | 0.015 | 4.80E-10 |  | 0.015 | 0.007 | 0.643 |
| Snoring | rs10878269 | T | C | 0.364 | 0.001 | 0.009 | 4.90E-18 |  | 0.017 | 0.054 | 0.001 |
| Snoring | rs11018434 | A | G | 0.483 | 0.001 | -0.006 | 2.80E-09 |  | 0.015 | -0.005 | 0.729 |
| Snoring | rs12119849 | A | G | 0.087 | 0.002 | 0.011 | 3.90E-09 |  | 0.019 | 0.010 | 0.621 |
| Snoring | rs13251292 | G | A | 0.411 | 0.001 | 0.007 | 1.50E-10 |  | 0.015 | 0.021 | 0.157 |
| Snoring | rs1374895 | T | C | 0.545 | 0.001 | -0.006 | 1.50E-08 |  | 0.015 | 0.025 | 0.086 |
| Snoring | rs180110 | A | G | 0.590 | 0.001 | 0.006 | 6.90E-10 |  | 0.015 | 0.009 | 0.579 |
| Snoring | rs183066 | T | C | 0.593 | 0.001 | 0.006 | 3.60E-08 |  | 0.015 | 0.015 | 0.317 |
| Snoring | rs2664303 | T | C | 0.423 | 0.001 | -0.007 | 3.10E-12 |  | 0.015 | 0.000 | 0.999 |
| Snoring | rs3862266 | A | G | 0.306 | 0.001 | 0.007 | 1.20E-10 |  | 0.018 | 0.015 | 0.391 |
| Snoring | rs4976269 | A | G | 0.329 | 0.001 | -0.007 | 4.50E-11 |  | 0.016 | -0.006 | 0.707 |
| Snoring | rs592333 | G | A | 0.444 | 0.001 | 0.009 | 7.90E-18 |  | 0.015 | 0.028 | 0.060 |
| Snoring | rs6099273 | T | C | 0.252 | 0.001 | 0.007 | 3.80E-08 |  | 0.016 | 0.015 | 0.351 |
| Snoring | rs6117259 | C | T | 0.187 | 0.001 | 0.008 | 6.40E-09 |  | 0.020 | 0.023 | 0.241 |
| Snoring | rs61597598 | A | G | 0.136 | 0.002 | 0.011 | 1.10E-12 |  | 0.023 | 0.041 | 0.067 |
| Snoring | rs62330353 | G | A | 0.205 | 0.001 | 0.007 | 3.50E-08 |  | 0.022 | -0.017 | 0.441 |
| Snoring | rs725861 | G | A | 0.188 | 0.001 | 0.009 | 9.70E-13 |  | 0.016 | 0.025 | 0.135 |
| Snoring | rs8108822 | T | C | 0.095 | 0.002 | -0.011 | 7.70E-11 |  | 0.026 | 0.031 | 0.246 |
| Snoring | rs853241 | T | C | 0.272 | 0.001 | 0.007 | 1.10E-08 |  | 0.017 | -0.021 | 0.215 |
| Snoring | rs9521988 | A | G | 0.342 | 0.001 | -0.006 | 3.80E-08 |  | 0.016 | 0.007 | 0.661 |
| Snoring | rs9912001 | T | C | 0.338 | 0.001 | 0.007 | 8.80E-10 |  | 0.016 | 0.005 | 0.775 |

EA: effect allele; OA: other allele; EAF: effect allele frequency; Se: standard error; p: P value
