# Supplementary material for: No bidirectional relationship between sleep phenotypes and risk of proliferative diabetic retinopathy: a two-sample Mendelian randomization study
Source: Sci Rep. 2024 Apr 26;14:9585. doi: 10.1038/s41598-024-60446-3 (PMC11053118; doi:10.1038/s41598-024-60446-3)
Supplement: Supplementary file 4 — Supplementary Information 4. [file 41598_2024_60446_MOESM4_ESM.docx]

**Supplementary Table 2.** SNPs are used as genetic instrumental variables for PDR.

|  | **Chr** | **SNP** | **OA** | **EA** | **Gene** | ***P* value** | **Beta** | **Se** | **EAF** | **VEP** | **F statistic** |
| --- | --- | --- | --- | --- | --- | --- | --- | --- | --- | --- | --- |
| 1 | 1 | rs2476601 | A | G | PTPN22 | 2.96E-13 | -0.147 | 0.020 | 0.835 | 0.0001 | 53.235 |
| 2 | 6 | rs200979 | G | A | HIST1H3J | 7.87E-14 | 0.158 | 0.021 | 0.147 | 0.0002 | 55.839 |
| 3 | 6 | rs150420498 | G | A | ZBTB9 | 4.07E-40 | 0.451 | 0.034 | 0.057 | 0.0005 | 175.766 |
| 4 | 6 | rs2495961 | G | A | GRM4 | 4.84E-10 | 0.092 | 0.015 | 0.484 | 0.0001 | 38.742 |
| 5 | 11 | rs3842727 | G | T | TH | 3.91E-13 | 0.127 | 0.018 | 0.775 | 0.0001 | 52.687 |
| 6 | 11 | rs10765567 | A | T | FAT3 | 1.16E-18 | -0.137 | 0.016 | 0.324 | 0.0002 | 77.766 |

EA: effect allele; OA: other allele; EAF: effect allele frequency; Se: standard error; VEP: Variance explained.
