# Supplementary material for: No bidirectional relationship between sleep phenotypes and risk of proliferative diabetic retinopathy: a two-sample Mendelian randomization study
Source: Sci Rep. 2024 Apr 26;14:9585. doi: 10.1038/s41598-024-60446-3 (PMC11053118; doi:10.1038/s41598-024-60446-3)
Supplement: Supplementary file 5 — Supplementary Information 5. [file 41598_2024_60446_MOESM5_ESM.docx]

**Supplementary Table 3.** Power calculation for MR analysis.

| **Exposure and outcome** | **Category** | **Cases** | **Controls** | **Total** | **Ratio** | **R2** | **OR** | ***P* value** | **Power value** |
| --- | --- | --- | --- | --- | --- | --- | --- | --- | --- |
| Chronotype | CV | 449734 | - | 449734 |  | 1.264 | 0.890 | 0.143 | 100% |
| Daytime napping | BV | 196895 | 255738 | 452633 | 0.770 | 0.788 | 1.104 | 0.691 | 100% |
| Daytime sleepiness | BV | 104786 | 347285 | 452071 | 0.302 | 0.233 | 1.407 | 0.473 | 100% |
| Insomnia | BV | 108357 | 345022 | 453379 | 0.314 | 0.265 | 1.499 | 0.181 | 100% |
| Long sleep duration | BV | 34184 | 305742 | 339926 | 0.112 | 0.055 | 0.502 | 0.671 | 100% |
| Morning person | BV | 252287 | 150908 | 403195 | 1.672 | 1.099 | 0.918 | 0.113 | 100% |
| Short sleep duration | BV | 106192 | 305742 | 411934 | 0.347 | 0.137 | 1.545 | 0.517 | 100% |
| Obstructive sleep apnea | BV | 16761 | 201194 | 217955 | 0.083 | 0.110 | 1.206 | 0.091 | 100% |
| Sleep duration | CV | 446118 | - | 446118 |  | 0.429 | 0.805 | 0.216 | 100% |
| Snoring | BV | 151836 | 255230 | 407066 | 0.595 | 0.204 | 4.087 | 0.014 | 100% |

CV: Continuous variable; BV: Binary variable; Total: Total sample size; Ratio: Ratio of cases to controls; R2: Coefficient of determination (R2) of exposure on genetic variants; OR: Causal effect (odds ratio, exp(β1)) per SD change in exposure; *P* value: Significance level.
