# Supplementary material for: No bidirectional relationship between sleep phenotypes and risk of proliferative diabetic retinopathy: a two-sample Mendelian randomization study
Source: Sci Rep. 2024 Apr 26;14:9585. doi: 10.1038/s41598-024-60446-3 (PMC11053118; doi:10.1038/s41598-024-60446-3)
Supplement: Supplementary file 6 — Supplementary Information 6. [file 41598_2024_60446_MOESM6_ESM.docx]

Supplementary Table 4. Heterogeneity and horizontal pleiotropy analyses between sleep phenotypes and PDR in UK Biobank subjects.

| **Outcome** | **Exposure** | **Methods** | **SNPs** | **Beta** | **Se** | ***P*** | **OR (95%CI)** | **Heterogeneity** | | |  | **Pleiotropy** | | |  | **M-P** |
| --- | --- | --- | --- | --- | --- | --- | --- | --- | --- | --- | --- | --- | --- | --- | --- | --- |
|  |  |  |  |  |  |  |  | **Q** | ***P*** | **I2** |  | **Inter**  **-cept** | **Se** | ***P*** |  |  |
| PDR | Insomnia | MR Egger | 38 | 1.097 | 0.715 | 0.134 | 2.995 (0.737-12.162) | 39.490 | 0.359 | 0.063 |  | -0.010 | 0.008 | 0.225 |  | 0.349 |
|  |  | Weighted median | 38 | 0.683 | 0.344 | 0.047 | 1.980 (1.008-3.889) |  |  |  |  |  |  |  |  |  |
|  |  | IVW | 38 | 0.265 | 0.243 | 0.275 | 1.304 (0.810-2.099) |  |  |  |  |  |  |  |  |  |
|  |  | Simple mode | 38 | 0.837 | 0.648 | 0.205 | 2.310 (0.648-8.229) |  |  |  |  |  |  |  |  |  |
|  |  | Weighted mode | 38 | 0.918 | 0.521 | 0.087 | 2.504 (0.901-6.955) |  |  |  |  |  |  |  |  |  |
|  | Sleep  duration | MR Egger | 63 | 0.016 | 0.773 | 0.984 | 1.016 (0.224-4.618) | 68.765 | 0.259 | 0.098 |  | -0.002 | 0.009 | 0.807 |  | 0.249 |
|  |  | Weighted median | 63 | -0.194 | 0.286 | 0.498 | 0.824 (0.471-1.442) |  |  |  |  |  |  |  |  |  |
|  |  | IVW | 63 | -0.168 | 0.192 | 0.382 | 0.846 (0.581-1.231) |  |  |  |  |  |  |  |  |  |
|  |  | Simple mode | 63 | -0.880 | 0.675 | 0.197 | 0.415 (0.110-1.558) |  |  |  |  |  |  |  |  |  |
|  |  | Weighted mode | 63 | -0.614 | 0.519 | 0.241 | 0.541 (0.196-1.496) |  |  |  |  |  |  |  |  |  |

PDR: proliferative diabetic retinopathy; IVW: inverse variance weighted; Q : Cochran’s Q value; *P*: P value; M-P: MR PRESSO Global Test P value.
